# Cerebrosides and Steroids from the Edible Mushroom *Meripilus giganteus* with Antioxidant Potential

**DOI:** 10.3390/molecules25061395

**Published:** 2020-03-19

**Authors:** András Sárközy, Zoltán Béni, Miklós Dékány, Zoltán Péter Zomborszki, Kinga Rudolf, Viktor Papp, Judit Hohmann, Attila Ványolós

**Affiliations:** 1 Department of Pharmacognosy, Interdisciplinary Excellence Centre, University of Szeged, Eötvös u. 6, H-6720 Szeged, Hungary; sarkozy@pharmacognosy.hu (A.S.); zombozope@pharmacognosy.hu (Z.P.Z.); 2 Spectroscopic Research Department, Gedeon Richter Plc., Gyömrői út 19-21, H-1103 Budapest, Hungary; z.beni@richter.hu (Z.B.); M.Dekany@richter.hu (M.D.); 3 Department of Plant Production and Plant Protection, University of Kaposvár, Guba S u. 40, H-7400 Kaposvár, Hungary; rukinga@freemail.hu; 4 Department of Botany, Szent István University, Villányi út 29-43, H-1118 Budapest, Hungary; Papp.Viktor@kertk.szie.hu; 5 Interdisciplinary Centre for Natural Products, University of Szeged, Eötvös u. 6, H-6720 Szeged, Hungary

**Keywords:** *Meripilus giganteus*, Meripilaceae, cerebroside, steroids, antioxidant, ORAC assay

## Abstract

The detailed chemical analysis of the methanol extract of *Meripilus giganteus* (Pers.) P. Karst. led to the isolation of two new cerebrosides, mericeramides A (**1**) and B (**2**) together with cerebroside B (**3**), ergosterol (**4**), 3β-hydroxyergosta-7,22-diene (**5**), cerevisterol (**6**), 3β-hydroxyergosta-6,8(14),22-triene (**7**), 3β-*O*-glucopyranosyl-5,8-epidioxyergosta-6,22-diene (**8**) and (11*E*,13*E*)-9,10-dihydroxy-11,13-octadecadienoic acid (**9**). The structures of the compounds were determined on the basis of NMR and MS spectroscopic analysis. Mericeramide A (**1**) is the first representative of halogenated natural cerebrosides. The isolated fungal metabolites **1**–**9** were evaluated for their antioxidant activity using the oxygen radical absorbance capacity (ORAC) assay. Compounds **2,** **5** and **9** proved to possess considerable antioxidant effects, with 2.50 ± 0.29, 4.94 ± 0.37 and 4.27 ± 0.05 mmol TE/g values, respectively. The result obtained gives a notable addition to the chemical and bioactivity profile of *M. giganteus*, highlighting the possible contribution of this species to a versatile and balanced diet.

## 1. Introduction

Mushrooms have long been valued not only as tasty and nutritional foods, but also as important tools of traditional medicine applied for treating several disease or alleviating symptoms. The edible polypore species, *Meripilus giganteus* (Pers.) P. Karst.—commonly known as “giant polypore”—a member of the family Meripilaceae [1], is characterized by its large-sized brownish-colored multi-capped fruiting body, which is mostly found close to stumps or on the roots of living broadleaved trees. Morphologically, the large compound basidiome of *M*. *giganteus* seems to be related to the well-known “Maitake” medicinal mushroom (*Grifola frondosa* (Dicks.) Gray), but the former is less grayish on the pileus, has smaller pores, and its pore surface becomes rapidly blackish when bruised or injured. The “giant polypore” was described by Persoon (1794) in Germany, under the name *Boletus giganteus* Pers. [2]; later this species was reported in several parts of Asia and North America suggesting that *M*. *giganteus* is widely distributed throughout the Northern Hemisphere [3]. However, a former taxonomic study queried the occurrence of *M*. *giganteus* in North America [4], which statement is also supported by the UNITE species hypothesis (SH) approach [5]. Consequently, former morphological observations and analyses of the available barcoding sequences deposited in public databases (e.g., GenBank, UNITE) indicate that the true “giant polypore” is a Eurasian species, with widespread distribution in Europe and toward Western Asia.

According to our search of the literature, several chemical and pharmacological studies have been carried out to identify the bioactive compounds of this species. Multiple studies revealed that *M. giganteus* contains several bioactive metabolites, including phenolic compounds, steroids, organic acids, tocopherols, and fatty acids [6]. Previously it has been demonstrated that extracts of *M. giganteus* possess notable antimicrobial and antioxidant properties [7,8]; moreover, a water-soluble glucan isolated from the sporocarps of giant polypore exhibited not only hydroxide and superoxide radical scavenging but also ferrous ion chelating activities [9]. Recently, Lenzi et al. demonstrated that the ethanol extract of the mushroom exhibits pro-apoptotic and anti-proliferative effects in leukemic cell lines [10]. The nutritional profile of giant polypore has also been investigated, which revealed high concentrations of carbohydrates and proteins, but a lower fat content [8].

In our search for bioactive fungal metabolites, we aimed to perform a detailed chemical examination of *M. giganteus* in order to identify its characteristic constituents.

## 2. Results and Discussion

**Figure 1:**
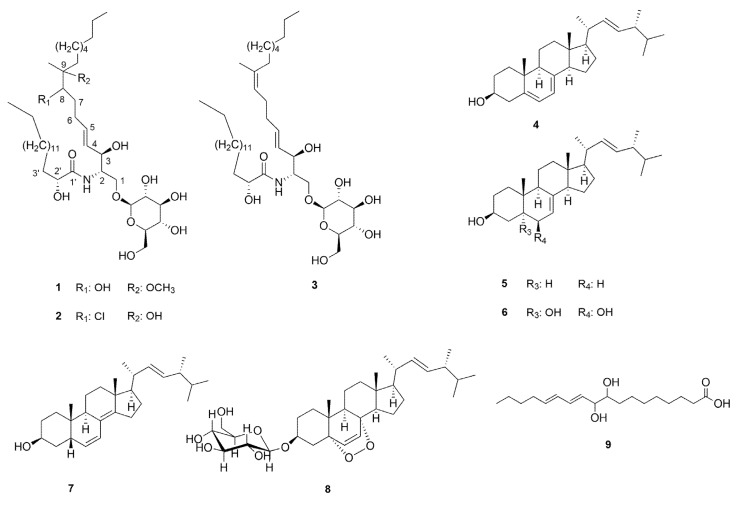
Compounds isolated from *M. giganteus* (stereodescriptors indicate only relative stereochemistry).

The mycochemical investigation of the methanol extract obtained from sporocarps of *M. giganteus* resulted in the identification of nine compounds (**1**–**9**) (Figure 1). The fungal extract was first subjected to solvent–solvent partition between aqueous MeOH and *n*-hexane, followed by extraction with chloroform. The resulted *n*-hexane and chloroform extracts were separated using a combination of flash column chromatography, preparative TLC and normal phase HPLC, to obtain compounds **1**–**9**. 

The consecutive analysis of the HRMS and NMR data led to the conclusion that **1** and **2** are new cerebrosides possessing the structures shown in Figure 1. These structural conclusions are based on the following arguments. 

HRMS data suggested elemental compositions of C_42_H_81_O_11_N and of C_41_H_79_ClO_10_N for **1** and **2**, respectively. The spectroscopic characteristics of **1** and **2** were very similar to those of cerebroside B (**3**) isolated from this species as well, and identified on the basis of NMR and MS data by comparing them to those reported earlier in the literature [11,12,13]. Apart from the resonances assigned to C7–C9 moiety, compounds **1** and **2** revealed highly similar spectral features in the ^1^H and ^13^C NMR spectra in comparison with those of **3**. The most significant differences were observed in the resonances assignable to the middle of the sphingadienine chain. Thus, instead of the resonances due to the double bond between C-8 and C-9 (5.14/125.0 (C-8), 136.9 (C-9)) in **3**, in compounds **1** and **2**, spectral features suggested the presence of a methine group (3.47/74.8 and 75.0 ppm in **1** and 3.74/70.8 ppm in **2**) and a quaternary carbon (at 80.2 and 75.8 ppm in the ^13^C NMR spectrum of **1** and **2**, respectively) both bound to heteroatoms. In parallel, the resonances assigned to C-19 methyl groups showed significant displacements both in **1** (1.06/18.6 ppm) and in **2** (1.20/22.4 ppm) compared to that in **3** (1.60/16.3 ppm). Furthermore, the additional 3H-singlet at 3.17 ppm in the ^1^H NMR spectrum of **1** (showing correlation with the 49.6 ppm ^13^C resonance in the HSQC spectrum), indicated the presence of an additional methoxy group in **1**. Based on these findings, and considering the elemental compositions and the observed HMBC correlations of H-6 with C-8, H-8 with C-6, C-10 and C-19 together with those of H-19 with C-8, C-9 and C-10 (see complete assignments in Table 1), 8-hydroxy-9-methoxy and 8-chloro-9-hydroxy cerebroside structures (Figure 1) were proposed for **1** and **2**, respectively.

Unfortunately, the absolute stereochemistry of **1** and **2** could not be determined from the collected NMR data. However, except C-8 and C-9 centers, the highly similar ^1^H and ^13^C NMR features of **1** and **2** in comparison with the appropriate values of **3** suggested similar stereochemistry for the chiral centers of the sugar moieties and the C-2, C-3 and C-2′ centers in all three compounds. Moreover, in the case of **1**, the ^13^C resonances assigned for the C4–C8 moiety were split into two peaks with a ca. 1:1 ratio. This suggested that **1** was isolated as a mixture of C-8 or/and C-9 epimers. Similar resonance splitting was described by Qi et al. [14] in the case of the 8,9-dihydroxy analogues reported as the oxidation products of cerebroside B, using OsO_4_ as the oxidizing agent. Interestingly, similar resonance splitting was not observed in case of **2**. 

Ergosterol (**4**) and 3β-hydroxyergosta-7,22-diene (**5**) were structurally identified by comparing the chromatographic and spectral characteristics to those of reference standards. Cerevisterol (**6**), 3β-hydroxyergosta-6,8(14),22-triene (**7**), and 3β-*O*-glucopyranosyl-5,8-epidioxyergosta-6,22-diene (**8**) and (11*E*,13*E*)-9,10-dihydroxy-11,13-octadecadienoic acid (**9**) reported here were structurally characterized on the basis of HRMS, MS-MS and standard one and two dimensional NMR data in comparison to those reported in the literature. Thus, the NMR spectroscopic data obtained for compounds **6**–**9** were found to be in good agreement with those reported earlier in another solvent [15,16,17]. Unfortunately, with the data at hand, the absolute stereochemistry of (11*E*,13*E*)-9,10-dihydroxy-11,13-octadecadienoic acid (**9**) could not be determined. In the case of 3β-hydroxyergosta-6,8(14),22-triene (**7**), the obtained ^1^H NMR chemical shifts and coupling constants were in excellent agreement with the reported fragmentary ^1^H NMR information [18]. The complete ^1^H and ^13^C NMR assignments for these already-reported compounds are provided in the Supplementary Materials, except for the ^1^H and ^13^C NMR assignments of **3**, which are listed in Table 1 to facilitate the discussion on the structural characterization of **1** and **2**.

The identified novel constituents (**1**–**2**) belong to the group of cerebrosides, classified as neutral glycosphingolipids which can be found in animals, plants, and fungi, the latter being particularly rich in such compounds. Though several halogenated natural products have been discovered in the last decades, compound **2** is the first member of halogenated cerebrosids. There is growing evidence, that several groups of organisms including fungi are producing halogenated organic compounds by their specific metabolism [19]. It is fairly improbable that chloroform known as the most frequently used halogenated non-polar solvent in extraction and isolation of natural products would serve as a halogenation agent; its distinctive stability makes chloroform an appropriate solvent for extractions and in organic reactions with a considerable limitation as reaction partner. Moreover, the close relative compound of chloroform, dichloromethane is extensively applied in pharmaceutical and food industry, for e.g. in the decaffeination of coffee and tea and in the extraction of different flavorings [20].

Cerebrosides originating from fungi are conserved structures which consist of a ceramide portion with 9-methyl-4,8-sphingadienine in amidic linkage to 2-hydroxyoctadecanoic or 2-hydroxyhexadecanoic acids, and a carbohydrate moiety, namely glucose or galactose. Several fungal cerebrosides were isolated from the plant pathogens *Fusarium graminearum* [21], *Fusarium solani* [22], but also from edible and/or medicinal mushrooms for, e.g., *Clitocybe geotropa* and *Clitocybe nebularis* [23], *Lentinus edodes* [24], *Polyporus squamosus* [25], and *Schizophyllum commune* [26].

The isolated compounds **1**–**9** were examined in the ORAC assay to explore their potential antioxidant properties (see Table 2). Among the identified fungal metabolites of *M. giganteus*, mericeramide B (**2**), 3β-hydroxyergosta-7,22-diene (**5**), and (11*E*,13*E*)-9,10-dihydroxy-11,13-octadecadienoic acid (**9**) demonstrated noteworthy antioxidant activity compared to the reference compound, ascorbic acid. Previous studies revealed that compound **3** had no ORAC and lipid peroxide inhibiting property, but exhibited a slight effect in 2,2,1-diphenyl-1-picrylhydrazyl (DPPH) and superoxide dismutase (SOD)-like activity assays [27,28]. Wei et al. investigated the antioxidant activity of compounds isolated from the medicinal mushroom *Hericium erinaceus* and experienced notable effects for cerevisterol (**6**) in the ORAC assay [29], while Athanasakis et al. found moderate DPPH activity for **6 [30]**. In another study cerevisterol (**6**) identified in the mycelia of *H. erinaceus* proved to possess considerable activity in the DPPH assay [31].

## 3. Materials and Methods

The chemicals used in the experiments were supplied by Sigma-Aldrich Hungary and Molar Chemicals, Hungary. Flash chromatography was carried out on a CombiFlash^®^ Rf+ Lumen instrument with integrated UV, UV-VIS and ELS detection using RediSep Rf Gold Normal Phase Silica Flash columns (4, 12 and 60 g) (Teledyne Isco, Lincoln, NE, USA). Normal-phase HPLC (NP-HPLC) separations were performed on a Wufeng LC-100 Plus HPLC instrument equipped with a UV-VIS detector (Shanghai Wufeng Scientific Instruments Co., Ltd., Shanghai, China) at 254 nm, using a Zorbax SILs column (250 × 4 mm, 5 µm; Agilent Technologies, Santa Clara, CA, USA). 

HRMS and MS-MS analyses were performed on a Thermo Velos Pro Orbitrap Elite (Thermo Fisher Scientific, Waltham, MA, USA) system. The ionization method was ESI-operated in negative or positive ion mode. The protonated (or deprotonated) molecular ion, as well as adduct ion peaks were fragmented by CID at a normalized collision energy of 35%–45%. For the CID experiment, helium was used as the collision gas. The samples were dissolved in methanol. Data acquisition and analysis were accomplished with Xcalibur software version 4.0 (Thermo Fisher Scientific).

NMR spectra were recorded at 298 K on a Bruker 500 (Bruker Corporation, Billerica, MA, USA) or a Varian 800 MHz (Varian, Inc., Palo Alto, CA, USA) NMR spectrometer, equipped with a liquid helium cooled 5 mm TCI CryoProbe or with a 5 mm HCN ^13^C-enhanced salt tolerant cold probe, respectively. CD_3_OD was used as a solvent in all cases. Chemical shifts were referenced to residual solvent signals (3.31 ppm for ^1^H and 49.15 for ^13^C). Standard one- (^1^H and ^13^C) and two-dimensional (COSY, HSQC, HMBC and ROESY) data were acquired in all cases, using the pulse sequences available in the Bruker Topspin 3.5 p7 or in the VNMRJ 3.2 sequence libraries. For data interpretation and reporting the ACD/Spectrus Processor 2017.1.3 software (ACDLabs, Toronto, ON, Canada) was used. 

### 3.1. Mushroom Material

Samples of *Meripilus giganteus* were collected from the roots of living beech and hornbeam in Mecsek Mts (Southern Transdanubia, Hungary) in September 2014 and 2015, near the villages of Püspökszentlászló (46°10′41″ N, 18°21′51″ E), Óbánya (46°13′08”N, 18°23′51”E), and Bakonya (46°05′54″ N, 18°03′56″ E), and in Visegrád Mts (Central Hungary), near the village of Pilismarót (47°44′19″ N, 18°50′43″ E) in August 2016. The samples were identified based on macro- and microscopic features. The different collections were combined for the preparative mycochemical experiment. Voucher specimens have been deposited in the mycological collection of the Hungarian Natural History Museum (BP 106949-51).

### 3.2. Extraction and Isolation

The fresh mushroom material (12 kg) was extracted with methanol (46 L) at room temperature. After concentration, the MeOH extract (145 g) was dissolved in 50% aqueous MeOH and subjected to solvent–solvent partition with *n*-hexane (5 × 400 mL). The *n*-hexane fraction (19.84 g) was subjected to flash chromatography on a silica gel column using a gradient system of *n*-hexane and acetone (0%–45%; t = 60 min). Fractions with similar compositions were combined according to TLC monitoring (A1-A15). The combined fraction A4 (1.8 g) was separated by flash chromatography using a mixture of *n*-hexane and acetone (0%–25%; t = 60 min), with increasing polarity, to obtain compounds **4** (0.35 g) and **5** (0.72 g). Fraction A6 (180 mg) was further separated by flash chromatography, applying an *n*-hexane–acetone solvent system (0%–30%; t = 45 min), and finally purified on normal phase HPLC using *n*-hexane–isopropanol (65:35) solvent system to obtain compound **6** (1.8 mg). The fractionation of fraction A6 (141 mg) on a normal phase column applying an *n*-hexane–isopropanol solvent system resulted in **1** (14.5 mg) and **2** (3.5 mg). 

The chloroform soluble phase (7.79 g) was subjected to flash chromatography in multiple steps on a silica gel column using a gradient system of chloroform–methanol (0–45%, t = 50 min) and *n*-hexane–acetone (0% to 100%, t = 60 min). Fractions with similar compositions were combined according to TLC monitoring (B1-B9). Fraction B4 (250 mg) was further separated by a combination of flash chromatography (*n*-hexane–acetone 5% to 25%, t = 50 min) and preparative TLC using an *n*-hexane–isopropanol (8:2) solvent system to obtain **7** (2.7 mg) and **9** (5.1 mg). Finally, the fractionation of B7 (185 mg) by normal phase flash chromatography led to the separation of **8** (15.2 mg) and **3** (25.9 mg).

Mericeramide A (**1**): white, amorphous solid; ^1^H and ^13^C-NMR data see in Table 1; HRESIMS: M + Na = 798.56859 (−2.0 ppm; C_42_H_81_O_11_NNa).

Mericeramide B (**2**): white, amorphous solid; ^1^H and ^13^C-NMR data see in Table 1; HRESIMS: M + Na = 802.51988 (−1.0 ppm; C_41_H_78_O_10_NClNa).

### 3.3. ORAC Assay

The ORAC assay was carried out on a 96-well microplate according to the method described by Mielnik et al. [32]. 20 µL of pure compounds of 0.01 mg/mL concentration were mixed with 60 µL of 2,2′-azobis(2-methyl-propionamidine)-dihydrochloride (AAPH) (12 mM final concentration) and 120 µL of fluorescein solution (70 nM final concentrations), then the fluorescence was measured over 3 h with 1.5-minute cycle intervals with a FlouStar Optima BMG Labtech plate-reader. All the experiments were carried out in triplicate; (±)-6-hydroxy-2,5,7,8-tetramethyl-chromane-2-carboxylic acid (Trolox) was used as standard. AAPH free radical and Trolox standard were purchased from Sigma-Aldrich (Budapest, Hungary). Fluorescein was purchased from Fluka analytical (Tokyo, Japan). The antioxidant capacity was expressed as mmol Trolox equivalent per g of compound (mmol TEg-1), with the help of GraphPad Prism 6.07.

## 4. Conclusion

The current study represents an in-depth chemical analysis of *Meripilus giganteus* and a valuable addition to future biological activity studies of this species. The combination of chromatographic methods led to the identification of nine compounds including two novel monoglycosylceramides, mericeramides A (**1**) and B (**2**). To the best of our knowledge, mericeramide A (**1**) is the first member of the halogenated natural cerebrosides. The ORAC assay conducted to examine the antioxidant properties of isolated metabolites **1**–**9** revealed that compounds **2, 5** and **9** possess considerable antioxidant effects. In this way, the edible giant polypore, containing fungal metabolites with antioxidant properties, has the potential for utilization as part of a healthy and varied diet.

## Figures and Tables

**Table 1:** ^1^H and ^13^C NMR assignments of **1**, **2** and **3** (CD_3_OD, 500 MHz (**1** and **2**) and 800 MHz (**3**)).

cmpd	1			2			3		
	^13^C	^1^H	mult. (*J*, Hz)	^13^C	^1^H	mult. (*J*, Hz)	^13^C	^1^H	mult. (*J*, Hz)
	(δ, ppm)			(δ, ppm)			(δ, ppm)		
1 a	69.8	4.10	dt (10.2, 6.1)	69.9	4.11	dt (10.3, 6.4)	69.9	4.12	m
1 b	69.8	3.72	dd (10.2, 3.5)	69.9	3.72	m	69.9	3.71	dd (10.4, 3.6)
2	54.8	3.99	m	54.7	4.00	m	54.7	3.99	m
3	73.1/73.1 *	4.17/4.17	t (7.0)	72.9	4.16	t (7.6)	73.0	4.13	m
4	131.19/131.29 *	5.53	m	132.3	5.54	dd (15.4, 7.2)	131.2	5.48	dd (15.5, 7.5)
5	134.95/135.04 *	5.76	m	133.7	5.72	dt (15.4, 7.2)	134.8	5.74	dtd (15.4, 6.6, 0.7)
6 a	31.0	2.06	m	31.4	2.11	m	28.8	2.07	m
6 b	31.0	2.31	m	31.4	2.39	m			
7 a	31.6	1.65	m	32.9	1.64	m	34.0	2.05	m
7 b	31.6	1.32	m	32.9	2.11	m			
8	74.8/75.0 *	3.47	dd (10.3, 5.5)	70.8	3.74	m	125.0	5.14	tq (6.7, 1.0)
9	80.2			75.6			136.9		
10 a	35.2	1.62	m	40.9	1.64	m	40.9	1.97	t (7.5)
10 b	35.2	1.49	m	40.9	1.59	m			
11	24.0	1.32	m	24.1	1.38	m	29.3	1.39	m
12–15′	30.9	1.30	m	30.9	1.29	m	30.5	1.29	m
16	33.2	1.28	m	33.2	1.29	m	33.2	1.31	m
17	23.9	1.32	m	24.0	1.32	m	23.9	1.31	m
18	14.6	0.90	t (6.9)	14.6	0.90	t (6.9)	14.6	0.90	t (7.1)
19	18.6	1.06	s	22.4	1.20	s	16.3	1.59	d (1.0)
20	49.6	3.17	s						
1′	177.4			177.4			177.3		
2′	73.2	3.99	m	73.2	3.99	m	73.2	3.98	m
3′ a	36.0	1.72	m	36.0	1.72	m	36.0	1.71	m
3′ b	36.0	1.55	m	36.0	1.55	m	36.0	1.55	m
4′	26.4	1.42	m	26.4	1.42	m	26.3	1.40	m
5′–13′	30.9	1.30	m	30.6	1.31	m	30.7	1.31	m
14′	33.2	1.28	m	33.2	1.29	m	33.2	1.29	m
15′	23.9	1.32	m	23.9	1.32	m	23.9	1.31	m
16′	14.6	0.90	t (6.9)	14.6	0.90	t (6.9)	14.6	0.90	t (7.1)
1″	104.9	4.27	d (7.9)	104.9	4.27	d (7.8)	104.9	4.26	d (7.8)
2″	75.1	3.19	t (8.3)	75.1	3.19	t (8.4)	75.1	3.19	dd (9.3, 7.8)
3″	78.0	3.35	m	78.1	3.36	m	78.0	3.35	m
4″	71.7	3.28	m	71.7	3.28	m	78.1	3.27	m
5″	78.1	3.28	m	78.1	3.27	m	71.7	3.27	m
6″ a	62.8	3.66	m	62.8	3.66	m	62.8	3.67	dd (11.9, 5.5)
6″ b	62.8	3.87	d (11.8)	62.8	3.87	d (11.8)	62.8	3.86	dd (11.9, 1.2)

* due to the presence of diastereomers.

**Table 2:** Antioxidant activity of compounds **1**–**9** isolated from *M. giganteus*.

Compound	ORAC Antioxidant Activity (mmol TE/g)
**1**	1.81 ± 0.34
**2**	**2.50** ± 0.29
**3**	1.69 ± 0.20
**4**	1.12 ± 0.06
**5**	**4.94** ± 0.37
**6**	1.94 ± 0.08
**7**	1.65 ± 0.03
**8**	1.90 ± 0.05
**9**	**4.27** ± 0.05
Ascorbic acid	**6.96** ± 0.57

## Supplementary Materials

Supplementary materials are available online: Figures S1–S36.
