# Supplementary material for: Cerebrosides and Steroids from the Edible Mushroom Meripilus giganteus with Antioxidant Potential
Source: Molecules. 2020 Mar 19;25(6):1395. doi: 10.3390/molecules25061395 (PMC7144561; doi:10.3390/molecules25061395)
Supplement: Supplementary file 1 [file molecules-25-01395-s001.pdf]

Cerebrosides and steroids from the edible mushroom *Meripilus giganteus* with antioxidant potential

András Sárközy[1], Zoltán Béni[2], Miklós Dékány[2], Zoltán Péter Zomborszki[1], Kinga Rudolf [3], Viktor Papp[4], Judit Hohmann[1,5], Attila Ványolós[1]*

[1] *Department of Pharmacognosy, Interdisciplinary Excellence Centre, University of Szeged, Eötvös u. 6, H-6720 Szeged, Hungary; sarkozy@pharmacognosy.hu, zombozope@pharmacognosy.hu*

[2] *Spectroscopic Research Department, Gedeon Richter Plc., Gyömrői út 19-21, H-1103 Budapest, Hungary; z.beni@richter.hu, M.Dekany@richter.hu*

[3]*Department of Plant Production and Plant Protection, University of Kaposvár, H–7400 Kaposvár, Guba S u. 40, Hungary;*

[4]*Department of Botany, Szent István University, H-1118 Budapest, Villányi út 29-43, Hungary;*

[5]*Interdisciplinary Centre for Natural Products, University of Szeged, Eötvös u. 6, H-6720 Szeged, Hungary; hohmann@pharm.u-szeged.hu*

*Correspondence: hohmann@pharm.u-szeged.hu (J.H.), vanyolosa@pharmacognosy.hu (A.V.); Tel.: +36-62-54-6455

TABLE OF CONTENTS

Spectra and spectral data on compound 1 4

Figure S1. HRESI-MS spectrum of compound **1** 5

Figure S2. MSMS spectrum of compound **1** 5

Figure S3. 500 MHz ^{1}H spectrum of compound **1** in MeOD 6

Figure S4. 125.7 MHz ^{13}C NMR spectrum of compound **1** in MeOD 6

Figure S5. 500 MHz COSY spectrum of compound **1** in MeOD 7

Figure S6. 500 MHz HMBC spectrum of compound **1** in MeOD 7

Figure S7. 500 MHz HSQC spectrum of compound **1** in MeOD 8

Figure S8. 500 MHz ROESY NMR spectrum of compound **1** in MeOD 8

Spectra and spectral data on compound 2. 9

Figure S9. HRESI-MS spectrum of compound **2** 10

Figure S10. MSMS spectrum of compound **2** 10

Figure S11. 500 MHz ^{1}H spectrum of compound **2** in MeOD 11

Figure S12. 125.7 MHz ^{13}C NMR spectrum of compound **2** in MeOD 11

Figure S13. 500 MHz COSY spectrum of compound **2** in MeOD 12

Figure S14. 500 MHz HMBC spectrum of compound **2** in MeOD 12

Figure S15. 500 MHz HSQC spectrum of compound **2** in MeOD 13

Spectra and spectral data on compound 3. 14

Figure S16. HR-ESI-MS spectrum of compound **3** 144

Figure S17. MSMS spectrum of compound **3** 155

Figure S18. 800 MHz ^{1}H spectrum of compound **3** in MeOD 155

Figure S19. 201 MHz ^{13}C NMR spectrum of compound **3** in MeOD 166

Figure S20. 800 MHz 1D-ZTOCSY spectrum of compound **3** in MeOD (H-3 triplet is enlarged) 166

Spectra and spectral data on compound 6 17

Figure S21. HR-ESIMS spectrum of compound **6** ....**Error! Bookmark not defined.**7

Figure S22. MSMS spectrum of compound **6** 18

Figure S23. 800 MHz ^{1}H spectrum of compound **6** in MeOD 18

Figure S24. 201 MHz ^{13}C NMR spectrum of compound **6** in MeOD 19

Spectra and spectral data on compound 7 20

Figure S25. HR-ESI MS spectrum of compound **7** 21

Figure S26. MSMS spectrum of compound **7** 21

Figure S27. 800 MHz ^{1}H spectrum of compound **7** in MeOD 22

Figure S28. 201 MHz ^{13}C NMR spectrum of compound **7** in MeOD 22

Spectra and spectral data on compound 8 23

Figure S29. HR-ESI MS spectrum of compound.. 24

Figure S30. MSMS spectrum of compound **8** 24

Figure S31. 800 MHz ^{1}H spectrum of compound **8** in MeOD. 25

Figure S32. 201 MHz ^{13}C NMR spectrum of compound **8** in MeOD 25

Spectra and spectral data on compound 9 266

Figure S33. HRESI-MS spectrum of compound **9** ....**Error! Bookmark not defined.**

Figure S34. MSMS spectrum of compound **9** 27

Figure S35. 800 MHz ^{1}H spectrum of compound **9** in MeOD ....**Error! Bookmark not defined.**7

Figure S36. 201.1 MHz ^{13}C NMR spectrum of compound **9** in MeOD. 28

Spectra and spectral data on compound 1

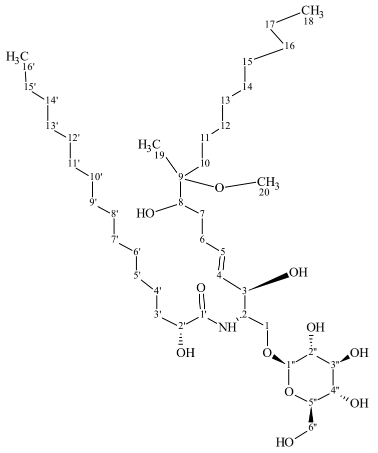

^{1}H and ^{13}C NMR assignments are given in Table 1.

HRMS: M+Na=798.56859 (delta=-2.0 ppm; $C_{42}H_{81}O_{11}NNa$). HR-ESI-MS-MS (CID=45%; rel. int. %): 780(9); 766(100); 636(18); 618(13); 604(8); 586(6); 572(4); 544(25); 512(10); 479(3); 392(5); 349(3).

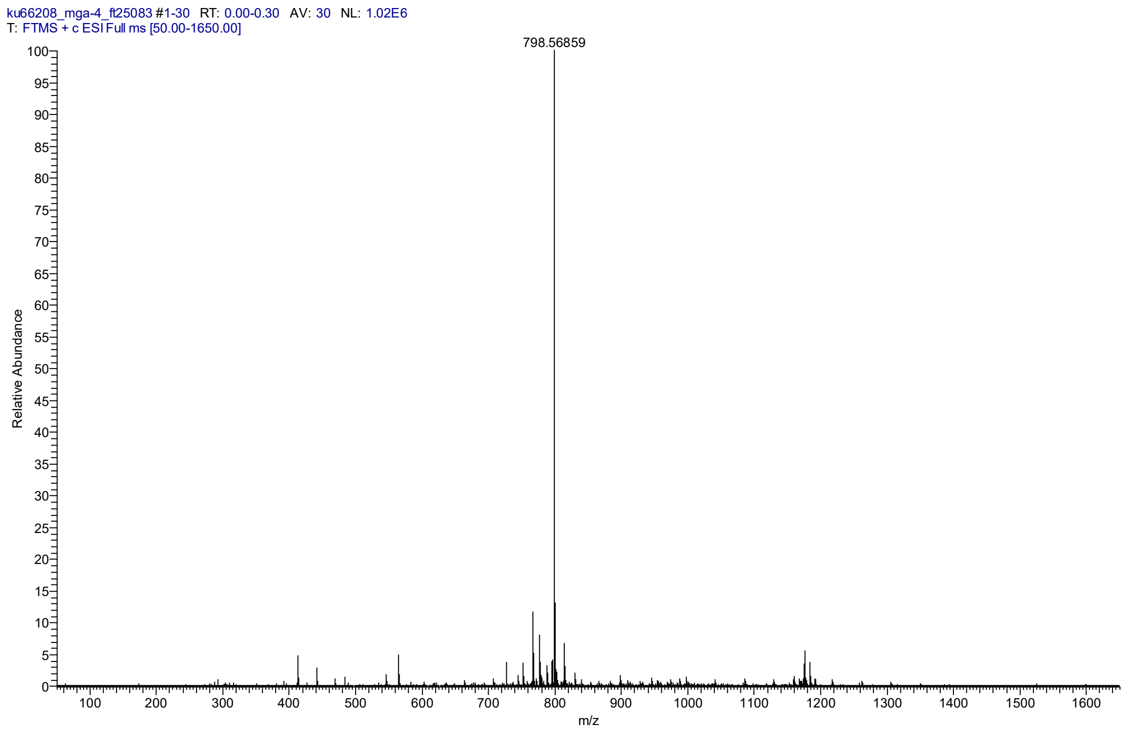

Figure S1. HRESI-MS spectrum of compound **1**.

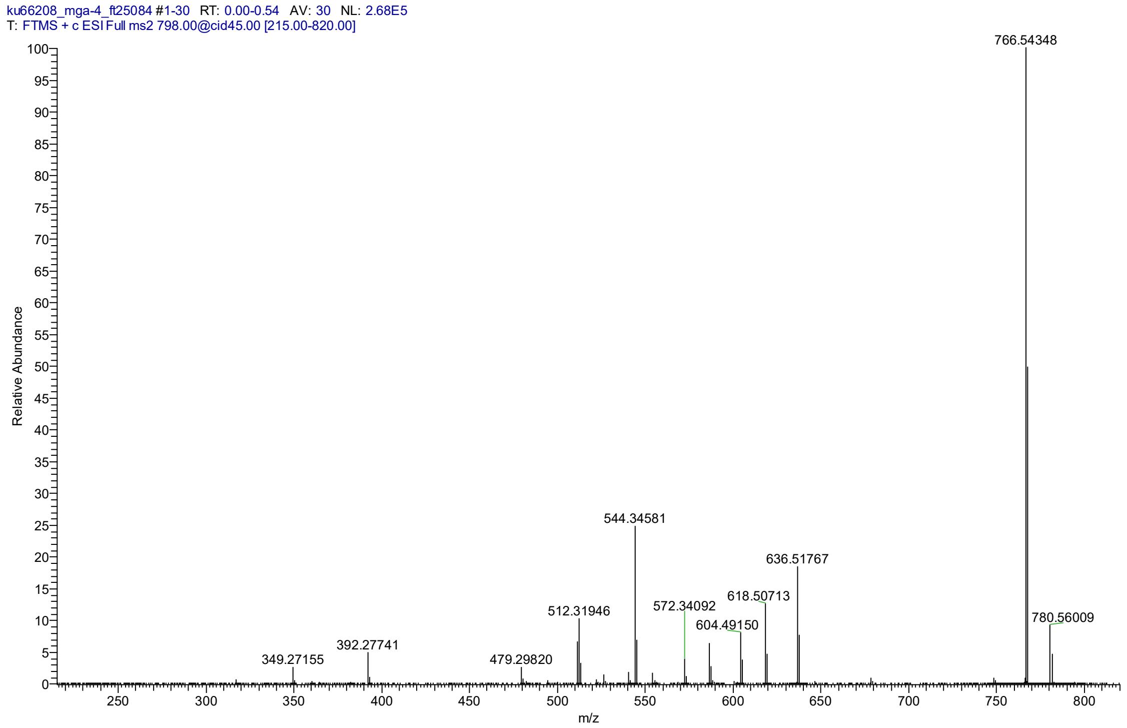

Figure S2. MSMS spectrum of compound **1**

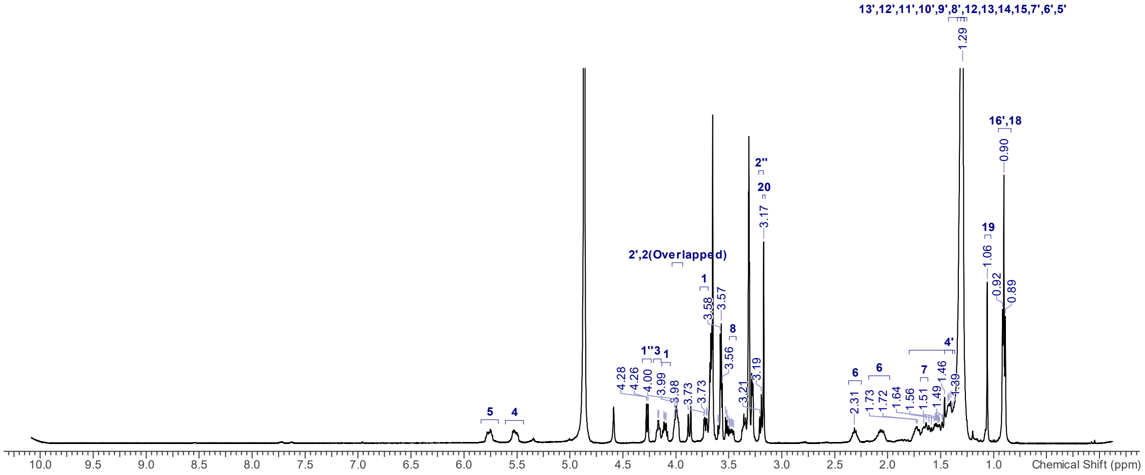

Figure S3. 500 MHz ^{1}H spectrum of compound **1** in MeOD

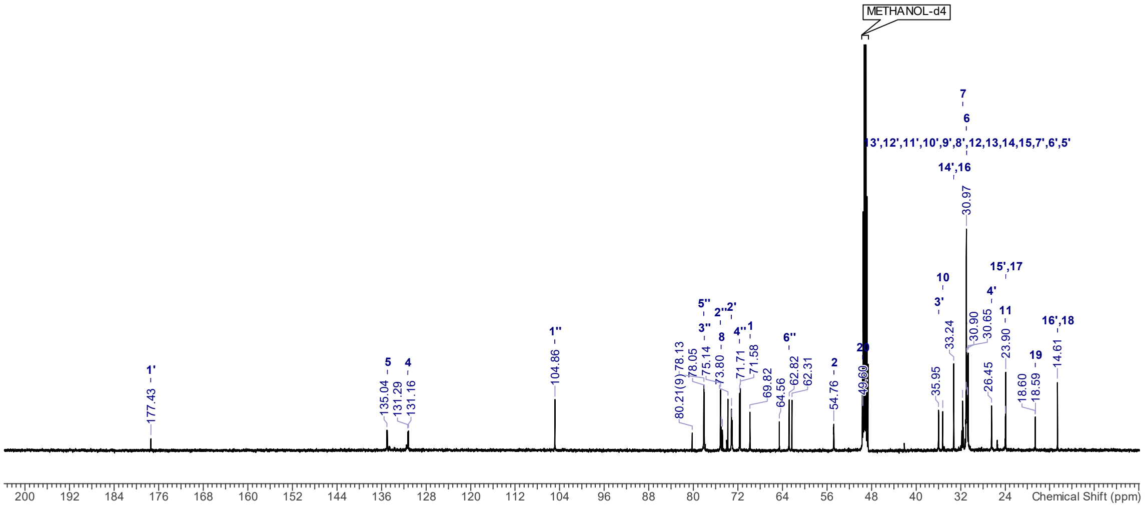

Figure S4. 125.7 MHz ^{13}C NMR spectrum of compound **1** in MeOD

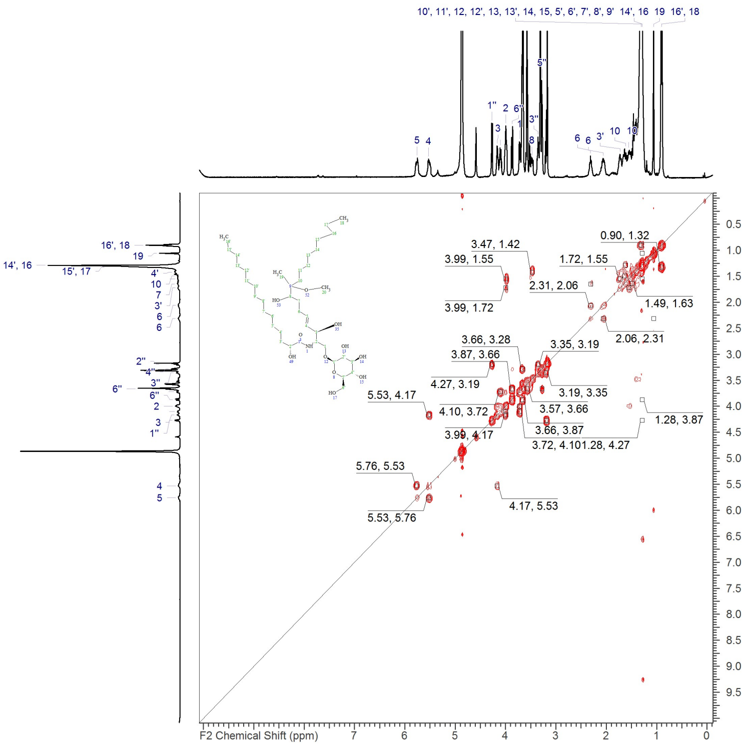

Figure S5. 800 MHz COSY spectrum of compound **1** in MeOD

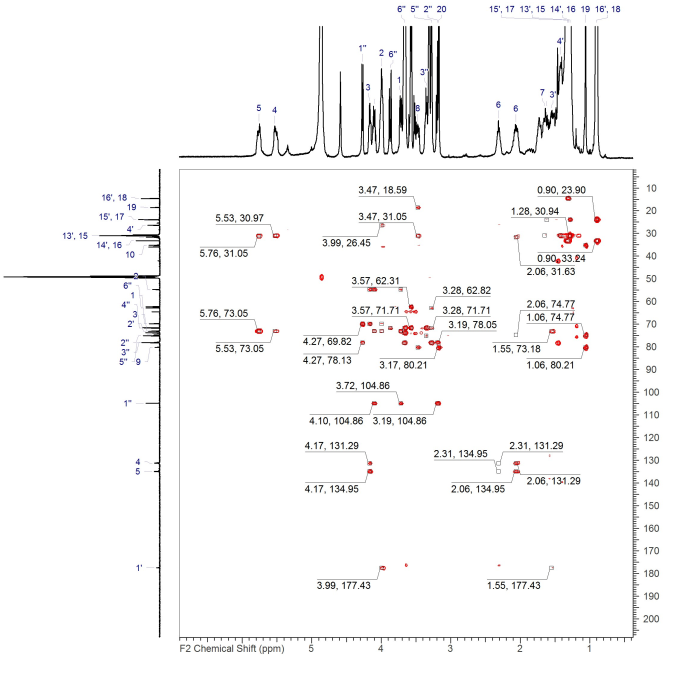

Figure S6. 800 MHz HMBC spectrum of compound **1** in MeOD

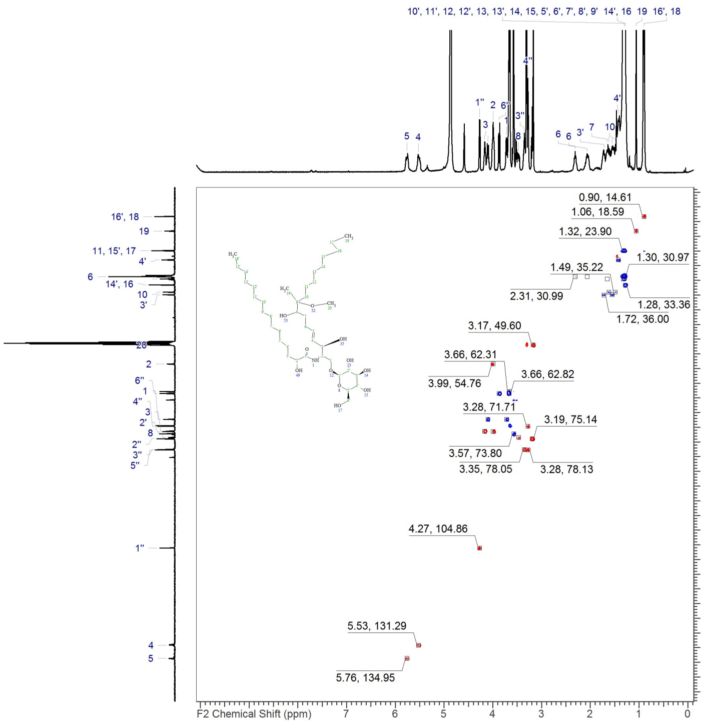

Figure S7. 800 MHz HSQC spectrum of compound **1** in MeOD

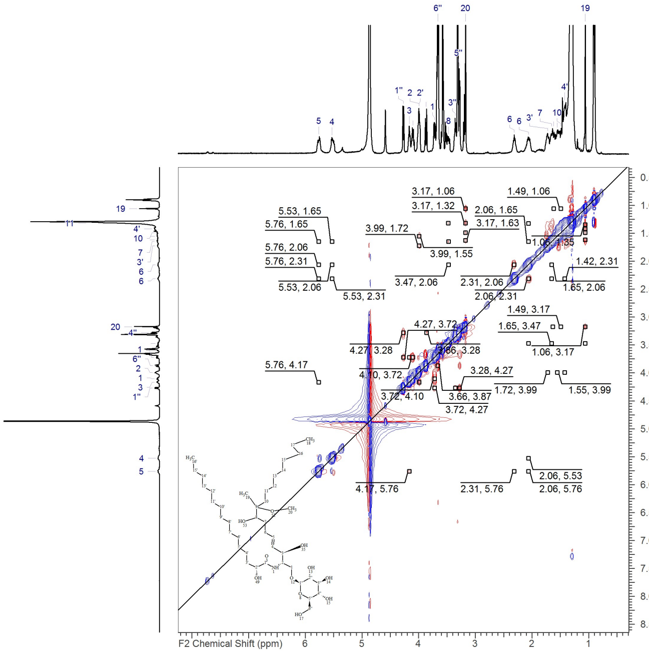

Figure S8. 800 MHz ROESY spectrum of compound **1** in MeOD

Spectra and spectral data on compound 2.

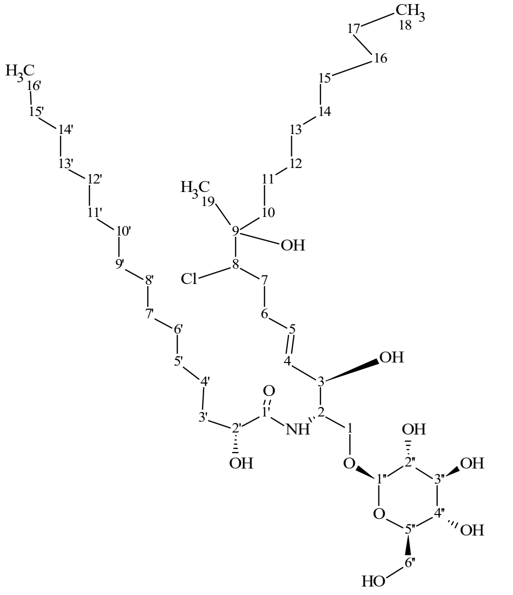

^{1}H and ^{13}C NMR data are given in Table 1

HRMS: M+Na=802.51988 (delta=-1.0 ppm; $C_{41}H_{78}O_{10}NClNa$). HR-ESI-MS-MS (CID=35%; rel. int. %): 766(100).

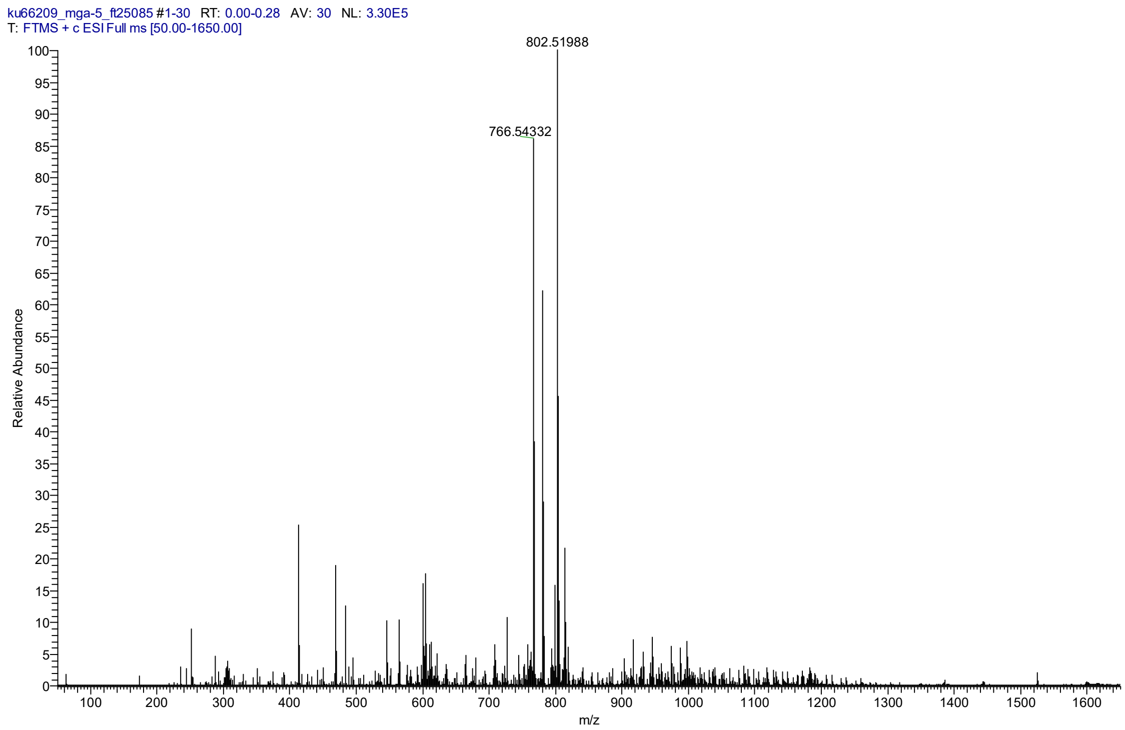

Figure S9. HRESI-MS spectrum of compound **2**

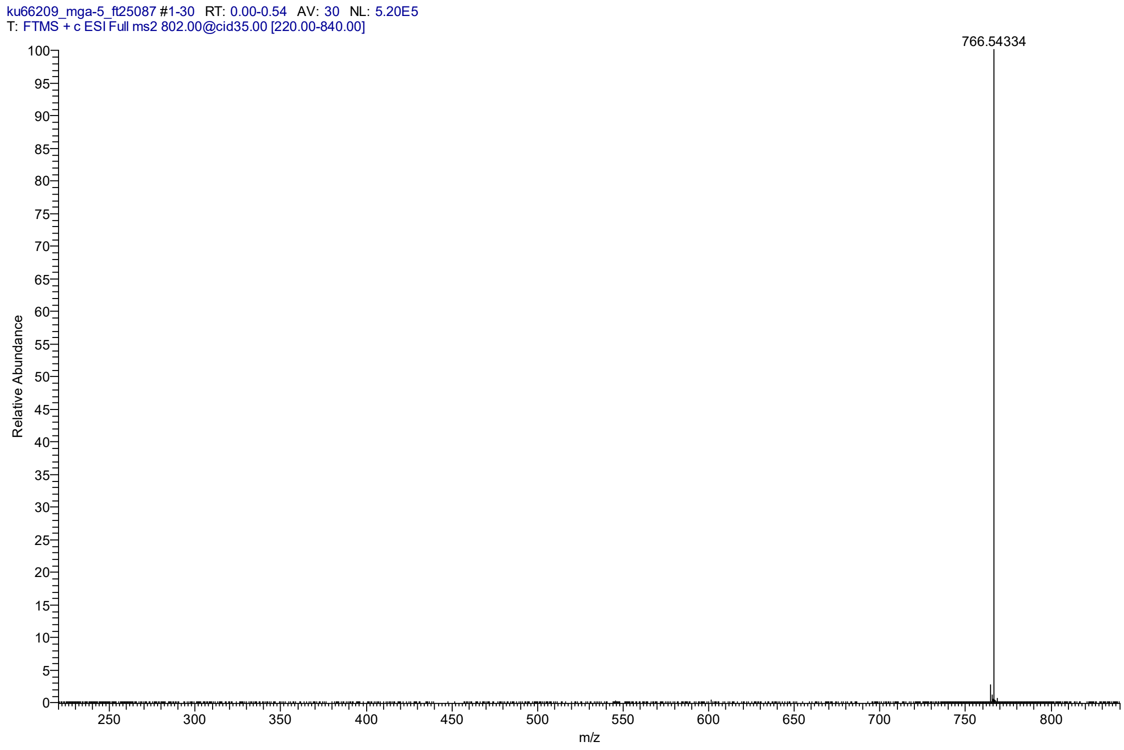

Figure S10. MSMS spectrum of compound **2**

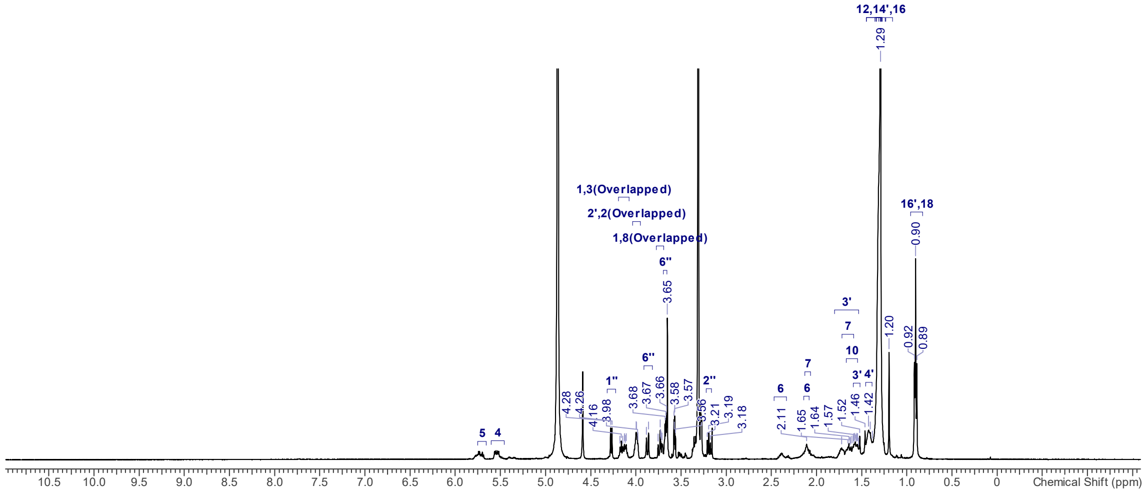

Figure S11. 500 MHz ^{1}H spectrum of compound **2** in MeOD

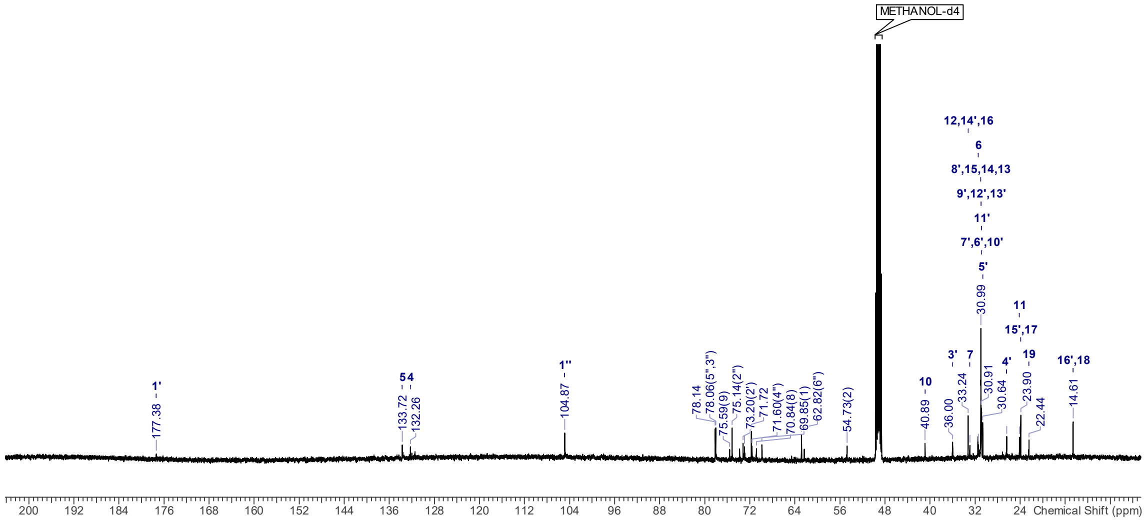

Figure S12. 125.7 MHz ^{13}C NMR spectrum of compound **2** in MeOD

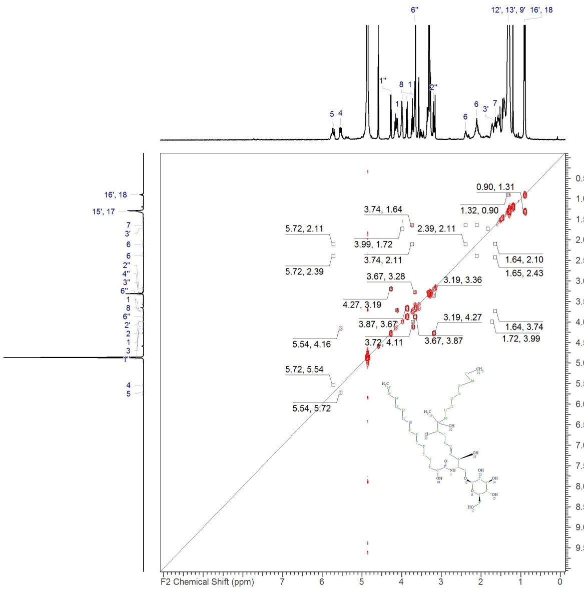

Figure S13. 500 MHz COSY spectrum of compound **2** in MeOD

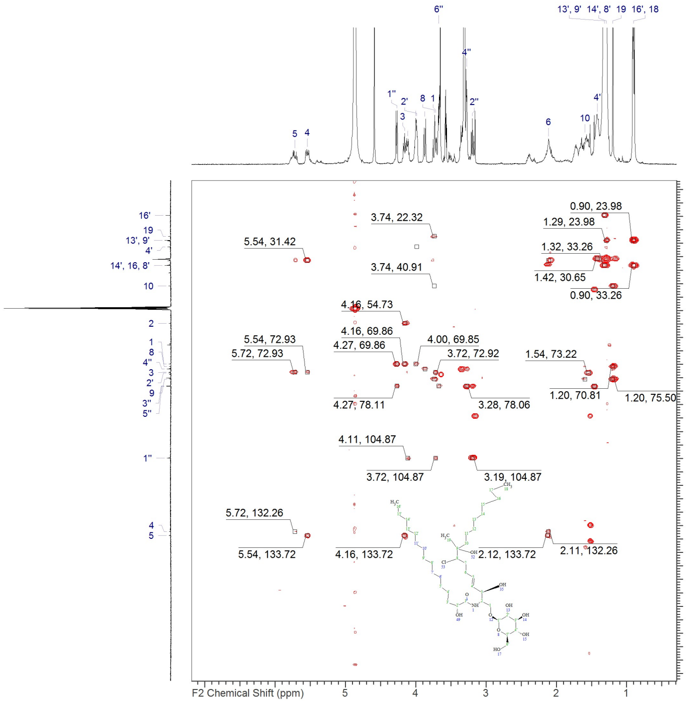

Figure S14. 500 MHz HMBC spectrum of compound **2** in MeOD

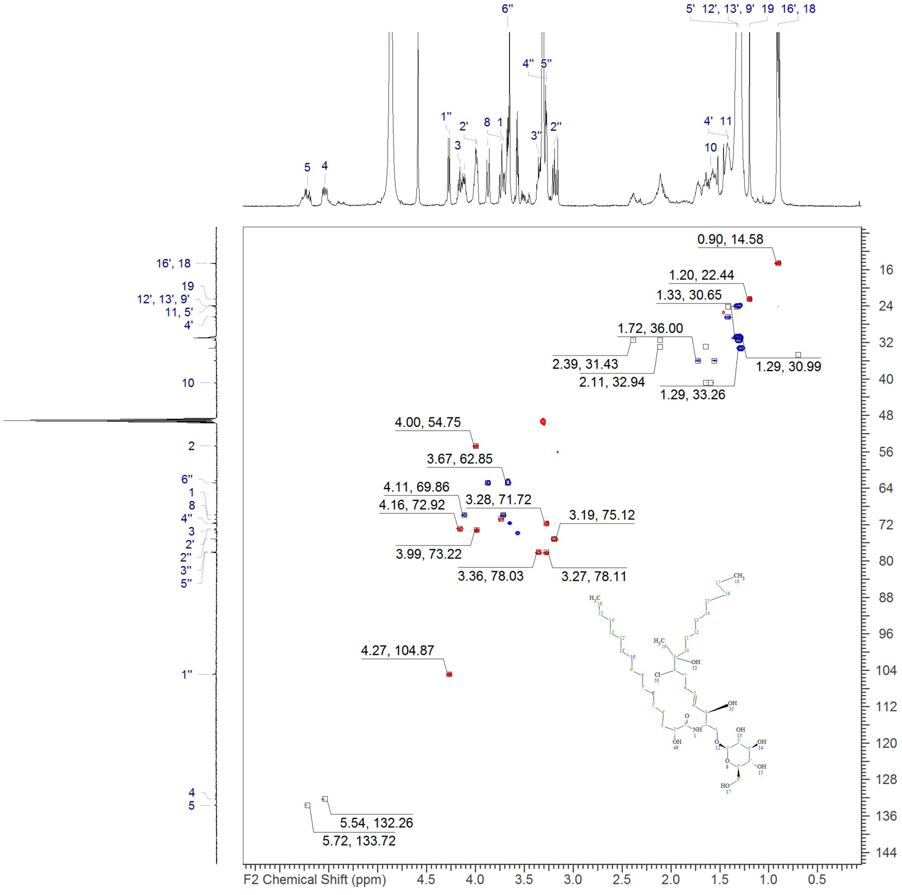

Figure S15. 500 MHz HSQC spectrum of compound **2** in MeOD

Spectra and spectral data on compound 3.

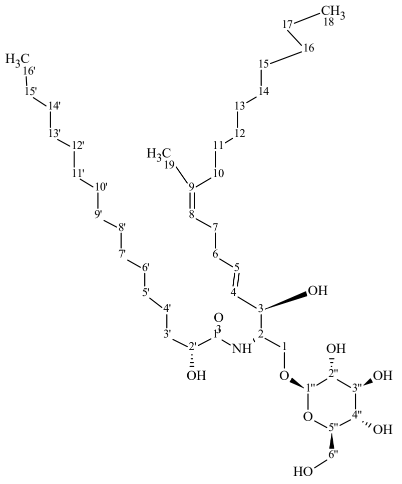

^1H and ^{13}C NMR data are shown in Table 1.

HRMS: M-H=726.55139 (delta=-1.6 ppm; $C_{41}H_{76}O_9N$). HR-ESI-MS-MS (CID=35%; rel. int. %): 565(51); 546(100).

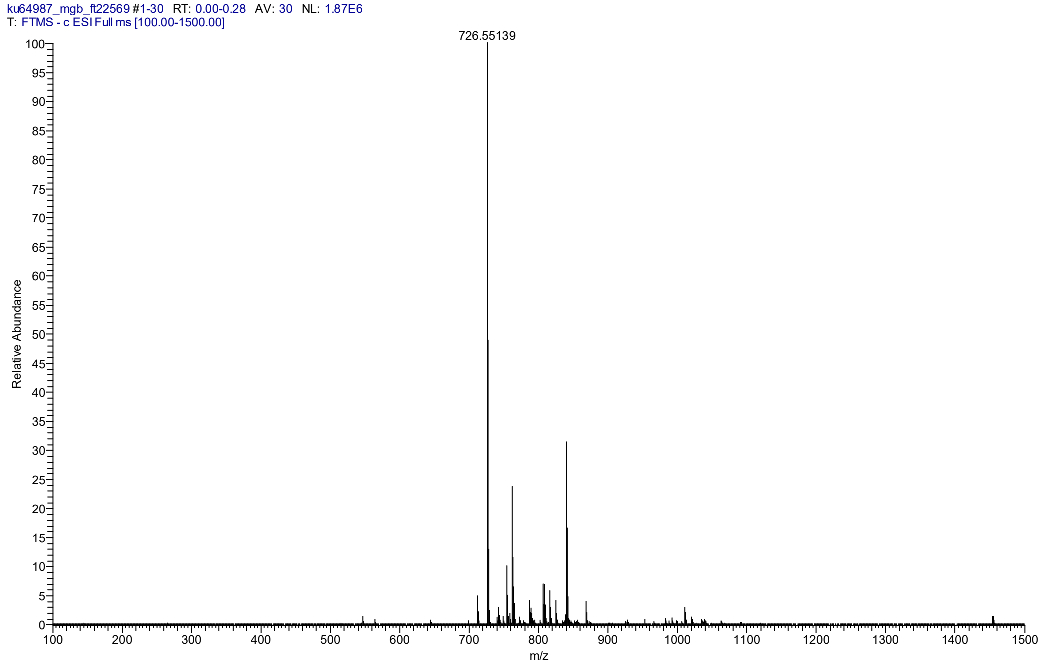

Figure S16. HR-ESI-MS spectrum of compound **3**

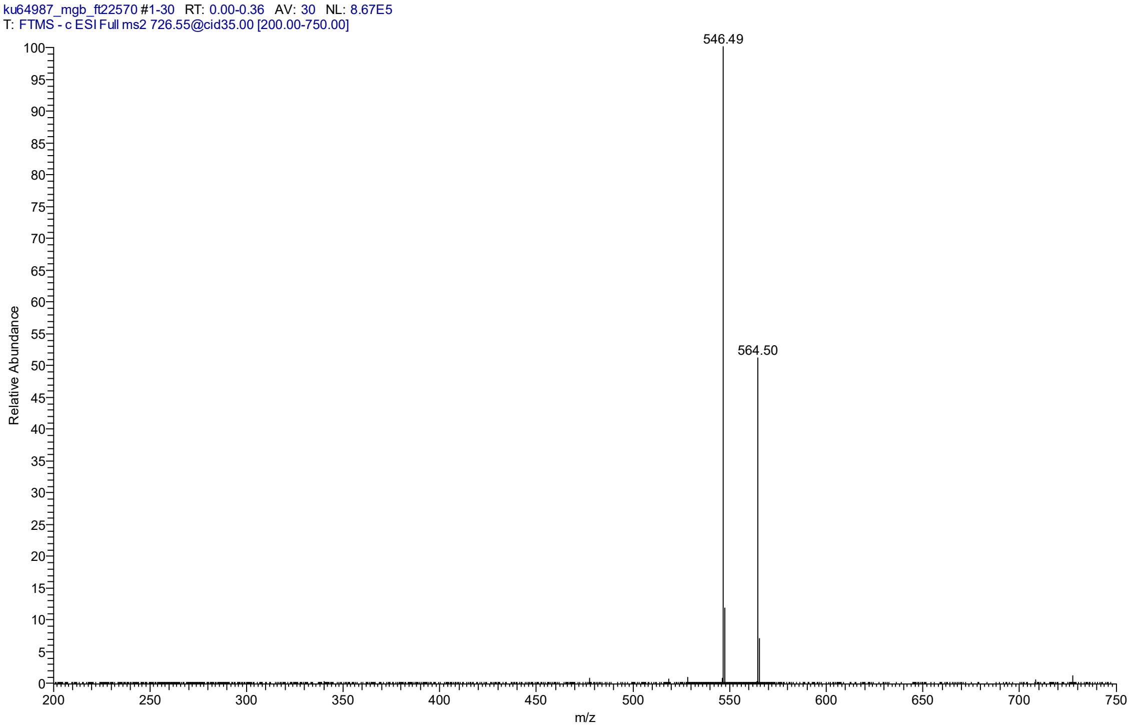

Figure S17. MSMS spectrum of compound **3**

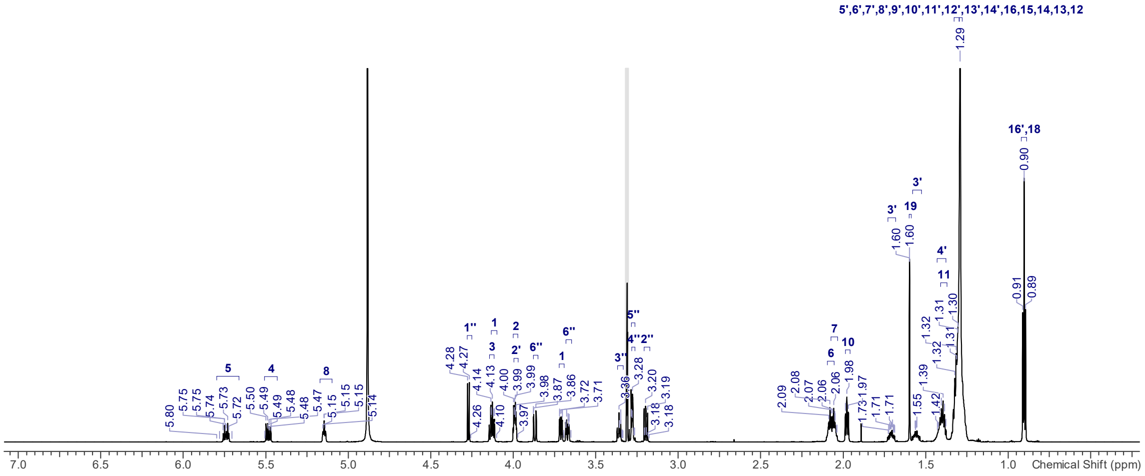

Figure S18. 800 MHz ^{1}H spectrum of compound **3** in MeOD

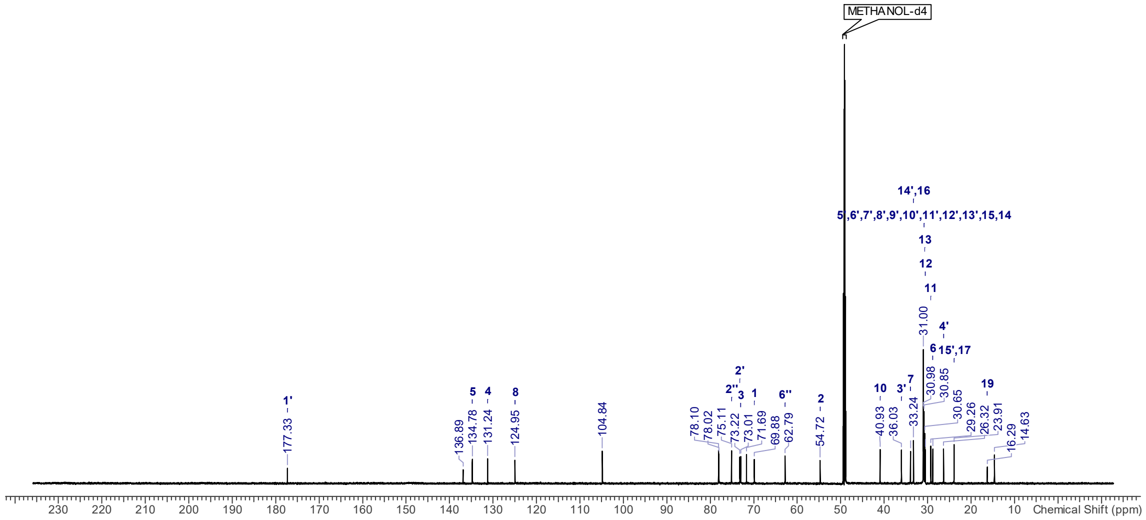

Figure S19. 201 MHz ^{13}C NMR spectrum of compound **3** in MeOD

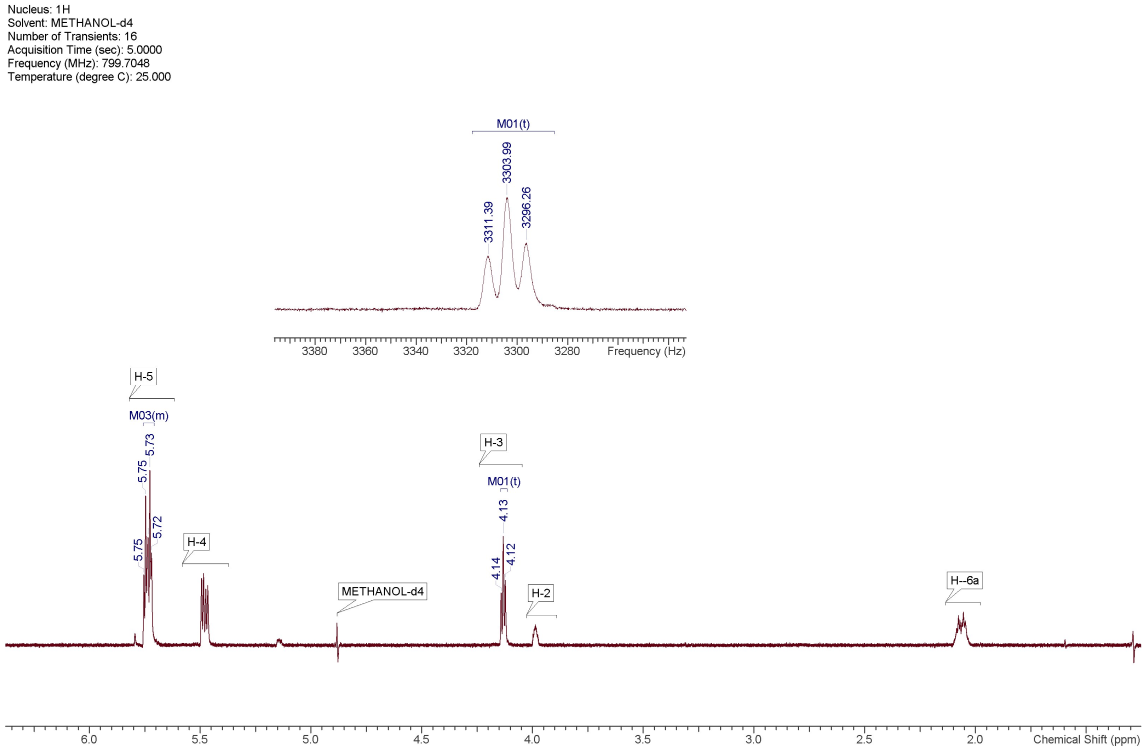

Figure S20. 800 MHz 1D-ZTOCSY spectrum of compound **3** in MeOD, H-3 triplet is enlarged

Spectra and spectral data on compound 6

^{1}H NMR (800 MHz, METHANOL-d_4) δ = 5.26 - 5.28 (1H, m, H-7), 5.17 - 5.25 (2H, m, H-22, 23), 3.98 (1H, tt, J = 11.4 Hz, J = 5.0 Hz, H-3), 3.55 (1H, dd, J = 4.9 Hz, J = 2.3 Hz, H-6), 2.10 (1H, dd, J = 13.3 Hz, J = 11.5 Hz, H-4), 2.04 - 2.07 (3H, m, H-9, 20, 33), 1.94 - 1.97 (1H, m, H-14), 1.82 - 1.89 (1H, m, H-24), 1.77 - 1.81 (2H, m, H-16, 2), 1.67 - 1.71 (1H, m, H-4), 1.61 - 1.64 (2H, m, H-1), 1.57 - 1.61 (3H, m, H-15, 11), 1.40 - 1.53 (4H, m, H-25, 15, 2, 1), 1.26 - 1.36 (3H, m, H-17, 16, 12), 1.06 (3H, s, H-19), 1.04 (3H, d, J = 6.7 Hz, H-21), 0.94 (3H, d, J = 6.8 Hz, H-28), 0.87 (3H, d, J = 6.7 Hz, H-26), 0.85 (3H, d, J = 6.7 Hz, H-27), 0.64 (3H, s, H-18)

^{13}C NMR (201 MHz, METHANOL-d_4) δ = 143.9 (C-8), 137.2 (C-22), 133.4 (C-23), 119.2 (C-7), 77.1 (C-5), 74.4 (C-6), 68.5 (C-3), 57.6 (C-17), 56.1 (C-14), 44.9 (C-13), 44.5, 44.5 (C-24, 9), 41.9 (C-20), 40.9 (C-12), 40.7 (C-4), 38.3 (C-10), 34.5 (C-25), 34.1 (C-1), 31.9 (C-2), 29.3 (C-16), 24.2 (C-15), 23.2 (C-11), 21.8 (C-21), 20.6 (C-26), 20.2 (C-27), 19.1 (C-19), 18.4 (C-28), 13.0 (C-18)

HRMS: M+H-H_2O-H_2O=395.33092 (delta=0.2 ppm; $C_{28}H_{43}O$). HR-ESI-MS-MS (CID=35%; rel. int. %): 377(100); 325(14); 311(88); 307(11); 293(21); 283(3); 269(16); 251(13); 241(3).

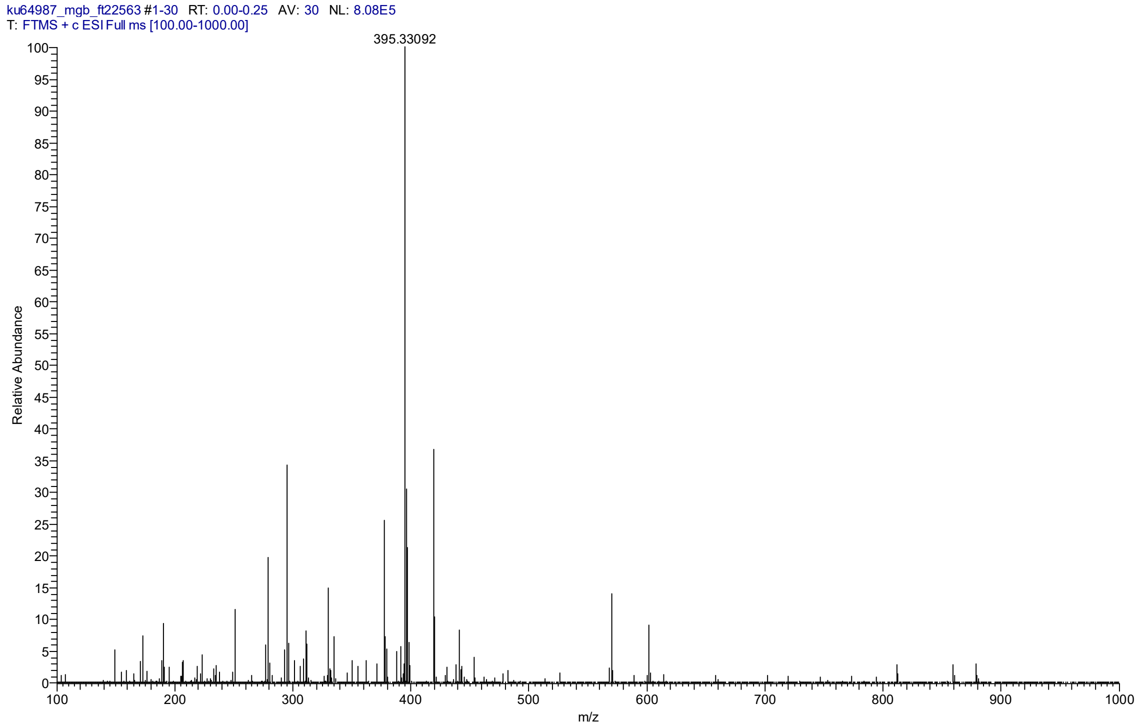

Figure S21. HR-ESIMS spectrum of compound **6**

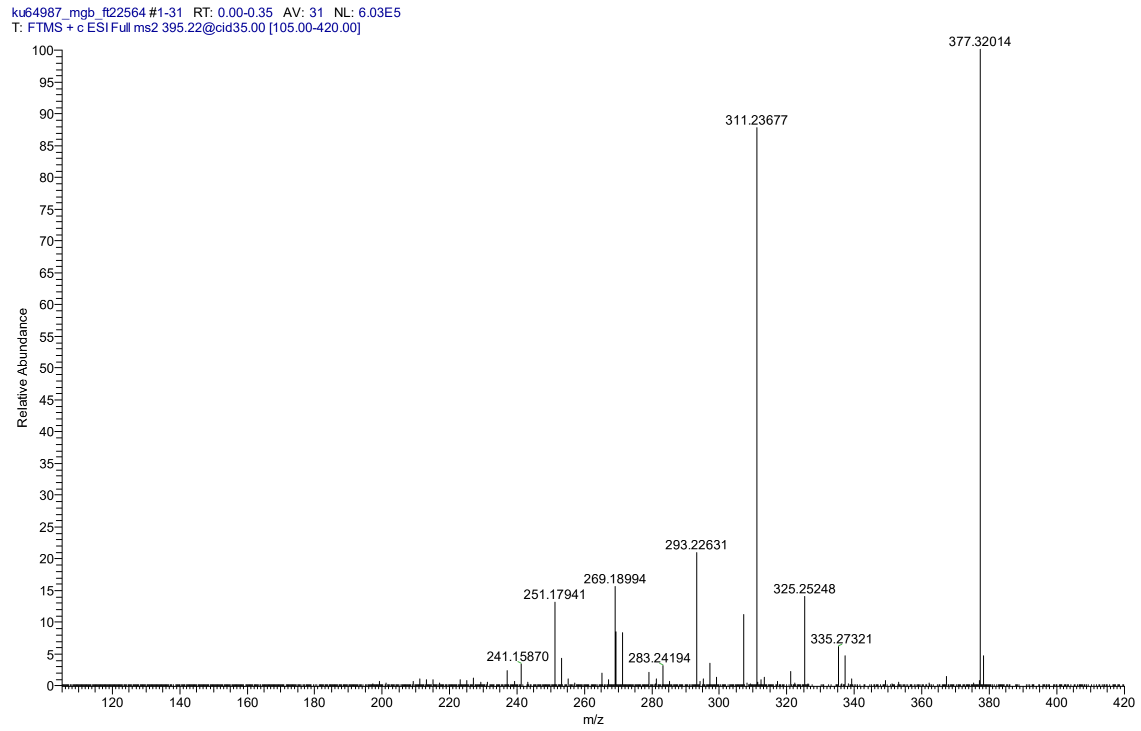

Figure S22. MSMS spectrum of compound **6**

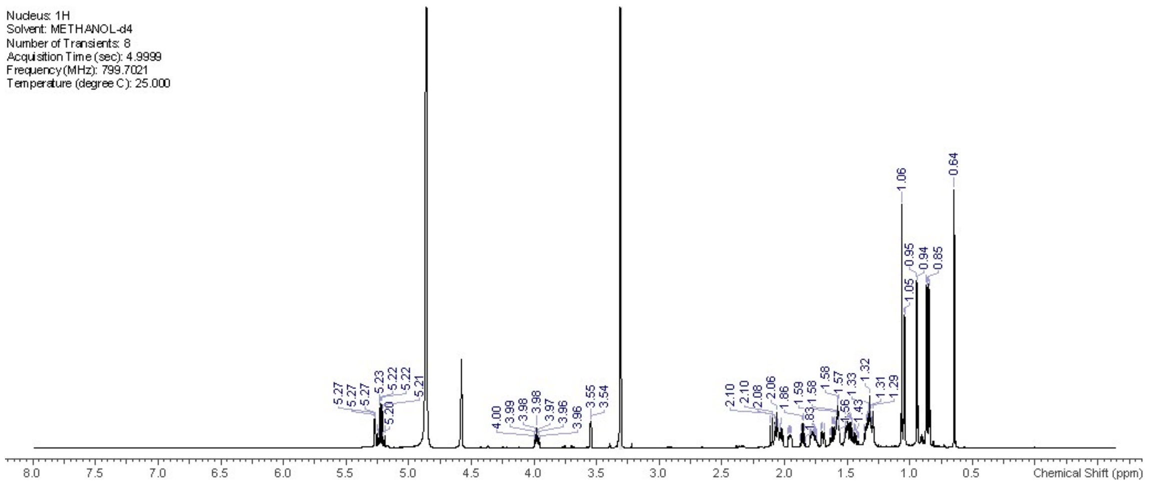

Figure S23. 800 MHz ^{1}H spectrum of compound **6** in MeOD

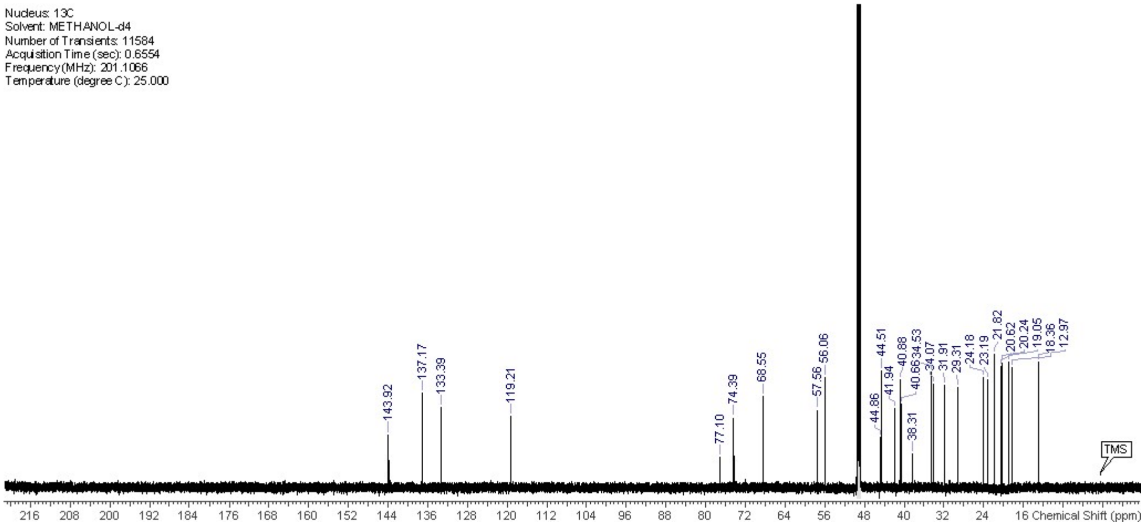

Figure S24. 201 MHz ^{13}C NMR spectrum of compound **6** in MeOD

Spectra and spectral data on compound 7

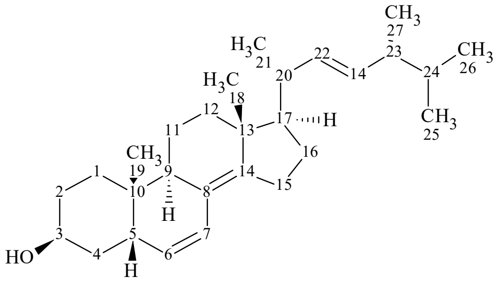

^{1}H NMR (800 MHz, METHANOL-d_4) δ = 6.06 (1H, d, J = 10.0 Hz, H-7), 5.50 (1H, dd, J = 9.9 Hz, J = 5.5 Hz, H-6), 5.19 - 5.29 (2H, m, H-14, 22), 3.97-4.03 (1H, m, H-3α), 2.44 - 2.53 (1H, m, H-9α), 2.32 - 2.39 (1H, m, H-15α), 2.20 - 2.28 (1H, m, H -15β), 2.10 - 2.18 (2H, m, H-5β, 20), 2.06 (1H, dt, J=12.6, 2.5 Hz, H-12β), 1.83 - 1.90 (1H, m, H-23), 1.75 - 1.80 (1H, m, H-16), 1.71 - 1.76 (1H, m, H-4), 1.41 - 1.65 (9H, m, H-24, 16, 11, 4, 2, 1), 1.34 - 1.40 (2H, m, H-12α), 1.18 - 1.23 (2H, m, H-17α), 1.06 (3H, d, J = 6.7 Hz, H-21), 0.95 (3H, d, J = 6.9 Hz, H-27), 0.93 s (3H, s, H-18), 0.87 (3H, d, J = 6.8 Hz, H-25), 0.85 (3H, d, J = 6.8 Hz, H-26), 0.75 (3H, s, H-19)

^{13}C NMR (201 MHz, METHANOL-d_4) δ = 147.5 (C-14), 137.0 (C-22), 133.5 (C-14), 131.3 (C-6), 126.8 (C-8), 125.5 (C-7), 67.0 (C-3), 57.7 (C-17), 44.7 (C-13), 44.5 (C-23), 41.0 (C-20), 40.6 (C-5), 38.8 (C-12), 37.5 (C-4), 35.7 (C-10), 35.7 (C-9), 34.5 (C-24), 29.4 (C-1), 29.3 (C-16), 28.8 (C-2), 25.9 (C-15), 23.7 (C-19), 20.6 (C-11)

HRMS: M+H=397.34657 (delta=0.2 ppm; $C_{28}H_{45}O$). HR-ESI-MS-MS (CID=35%; rel. int. %): 379(100); 369(3); 339(3); 327(7); 313(43); 295(19); 285(3); 271(8); 257(4); 239(3).

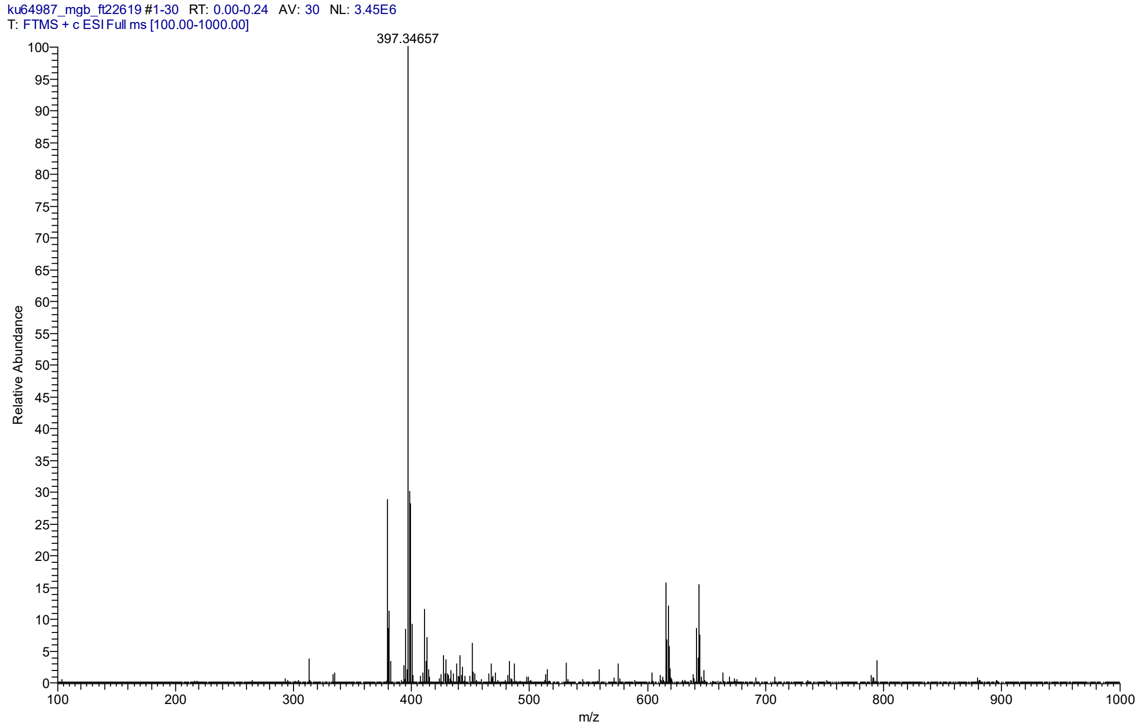

Figure S25. HR-ESI MS spectrum of compound **7**

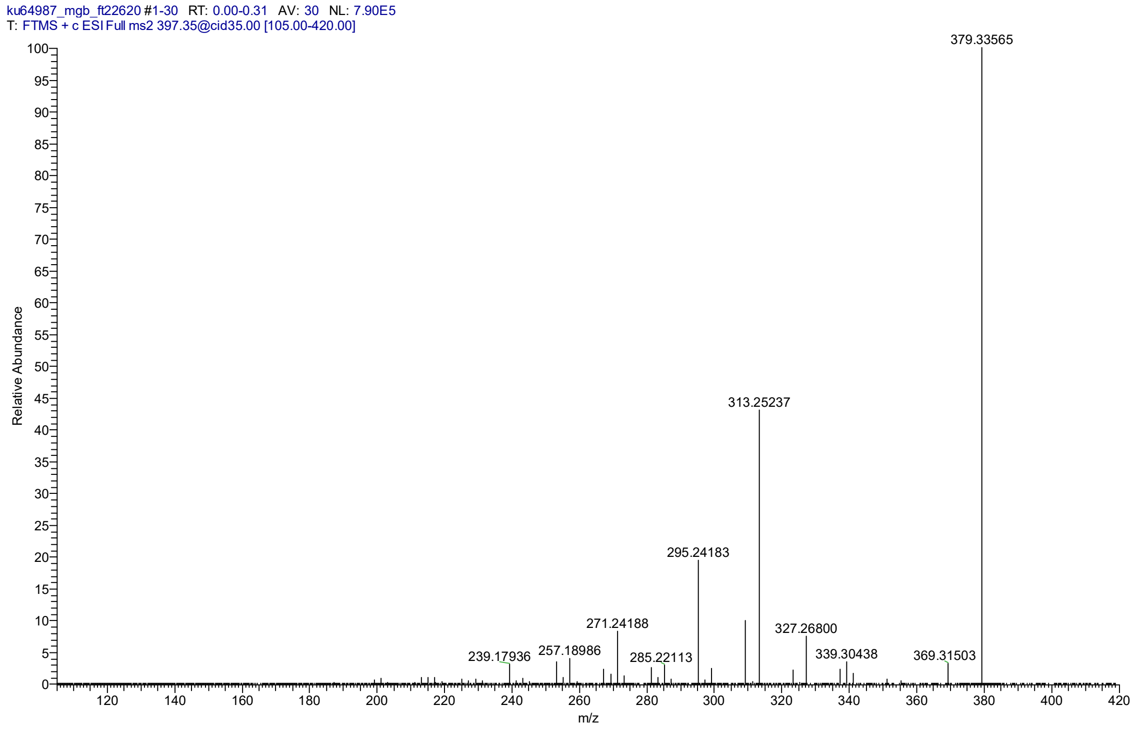

Figure S26. MSMS spectrum of compound **7**

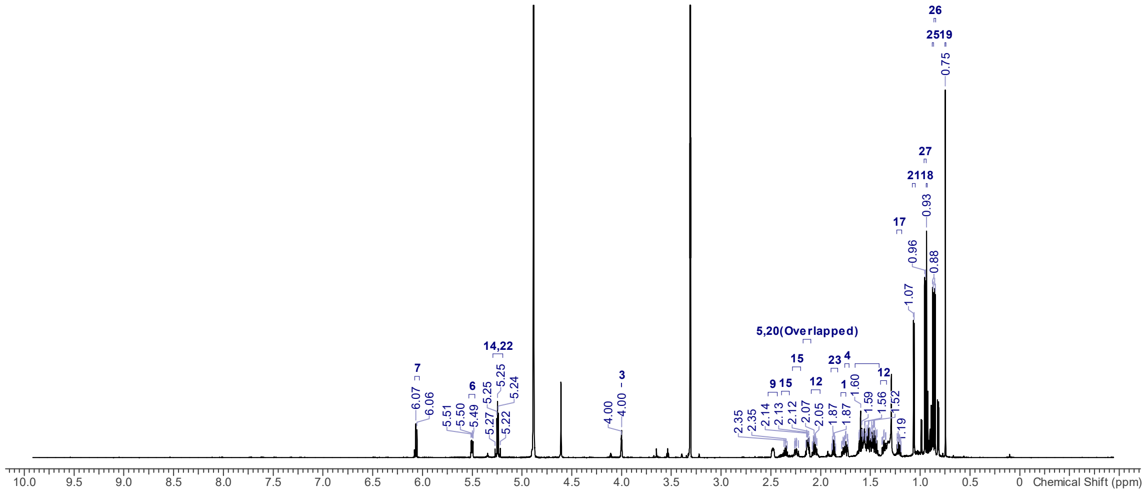

Figure S27. 800 MHz 1H spectrum of compound **7** in MeOD

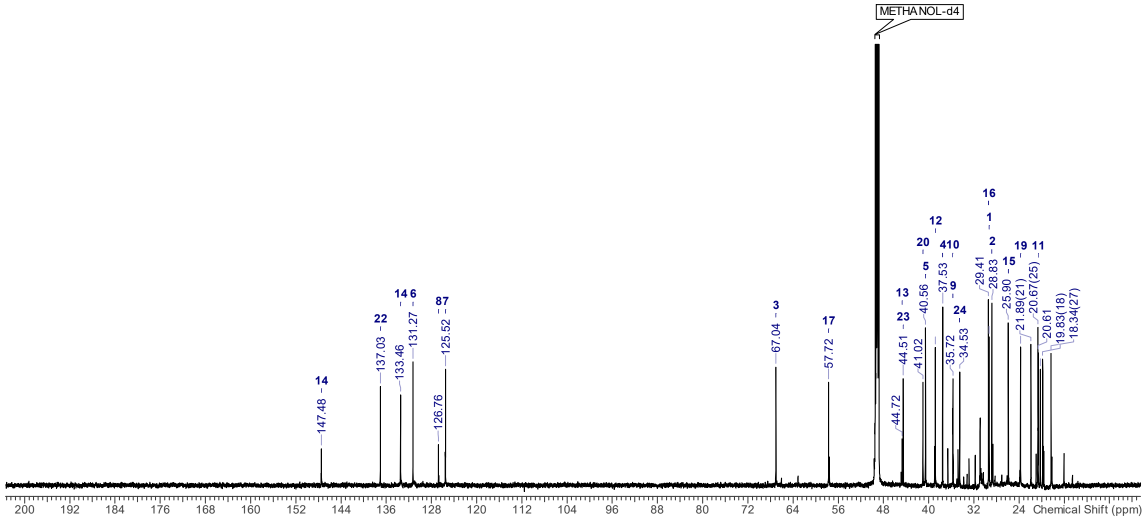

Figure S28. 201 MHz ^{13}C NMR spectrum of compound **7** in MeOD

Spectra and spectral data on compound 8

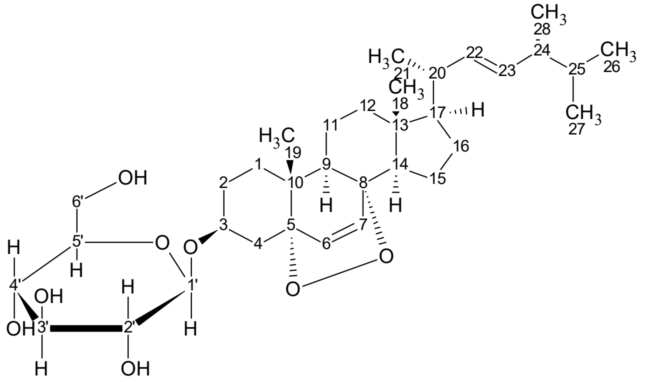

^{1}H NMR (800 MHz, METHANOL-d4) δ = 6.53 (1H, d, J = 8.6 Hz, H-7), 6.27 (1H, d, J = 8.5 Hz, H-6), 5.15 - 5.27 (1H, m, H-23, 22), 4.32 (1H, d, J = 7.8 Hz, H-1'), 3.91 (1H, ddd, J = 16.6 Hz, J = 11.6 Hz, J = 5.0 Hz, H-3), 3.84 (1H, dd, J = 11.9 Hz, J = 2.2 Hz, H-6'), 3.65 (1H, dd, J = 11.7 Hz, J = 5.1 Hz, H-6'), 3.31 - 3.35 (1H, m, H-3'), 3.23 - 3.31 (2H, m, H-5', 4'), 3.12 (1H, dd, J = 9.2 Hz, J = 7.8 Hz, H-2'), 2.12 - 2.17 (1H, m, H-4), 1.81 - 2.11 (6H, m, H-2, 4, 1, 12, 20, 24), 1.60 - 1.79 (3H, m, H-16, 2, 1), 1.48 - 1.58 (1H, m, H-15, 14, 11), 1.44 - 1.50 (1H, m, H-25), 1.40 - 1.47 (2H, m, H-11, 9), 1.35 - 1.44 (1H, m, H-16), 1.20 - 1.28 (3H, m, H-17, 15, 12), 1.02 (3H, d, J = 6.6 Hz, H-21), 0.93 (3H, d, J = 6.8 Hz, H-25), 0.91 (3H, s, H-19), 0.85 (3H, s, H-18), 0.86 (3H, d, J = 6.7 Hz, H-26), 0.84 (3H, d, J = 6.8 Hz, H-27)

^{13}C NMR (201 MHz, METHANOL-d4) δ = 136.9 (C-7), 136.9 (C-22), 133.6 (C-23), 131.9 (C-6), 103.1 (C-1'), 78.2 (C-3'), 78.0 (C-5'), 75.4 (C-3), 75.2 (C-2'), 71.7 (C-4'), 62.8 (C-6'), 57.7 (C-17), 53.2 (C-14), 52.9 (C-9), 44.5 (C-24), 41.3 (C-20), 40.8 (C-12), 36.1 (C-1), 35.0, 34.5 (C-25, 4), 30.9, 30.0 (C-16), 29.5 (C-2), 24.6 (C-15), 21.8, 21.7 (C-11), 21.5 (C-21), 20.6 (C-26), 20.2 (C-27), 18.7 (C-19), 18.3 (C-28), 13.4 (C-18)

HRMS: M+Na=613.37103 (delta=-0.1 ppm; $C_{34}H_{54}O_8Na$). HR-ESI-MS-MS (CID=35%; rel. int. %): 595(100); 585(7); 584(4); 580(5); 493(4); 451(74); 433(31); 393(2).

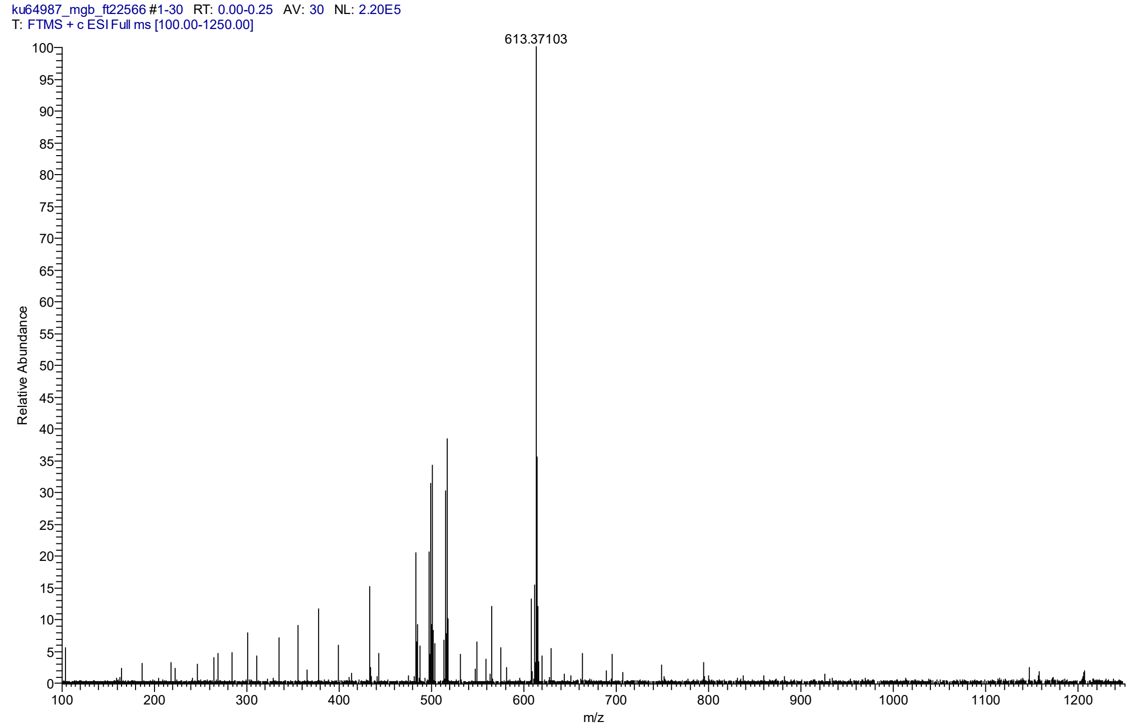

Figure S29. HR-ESI MS spectrum of compound **8**

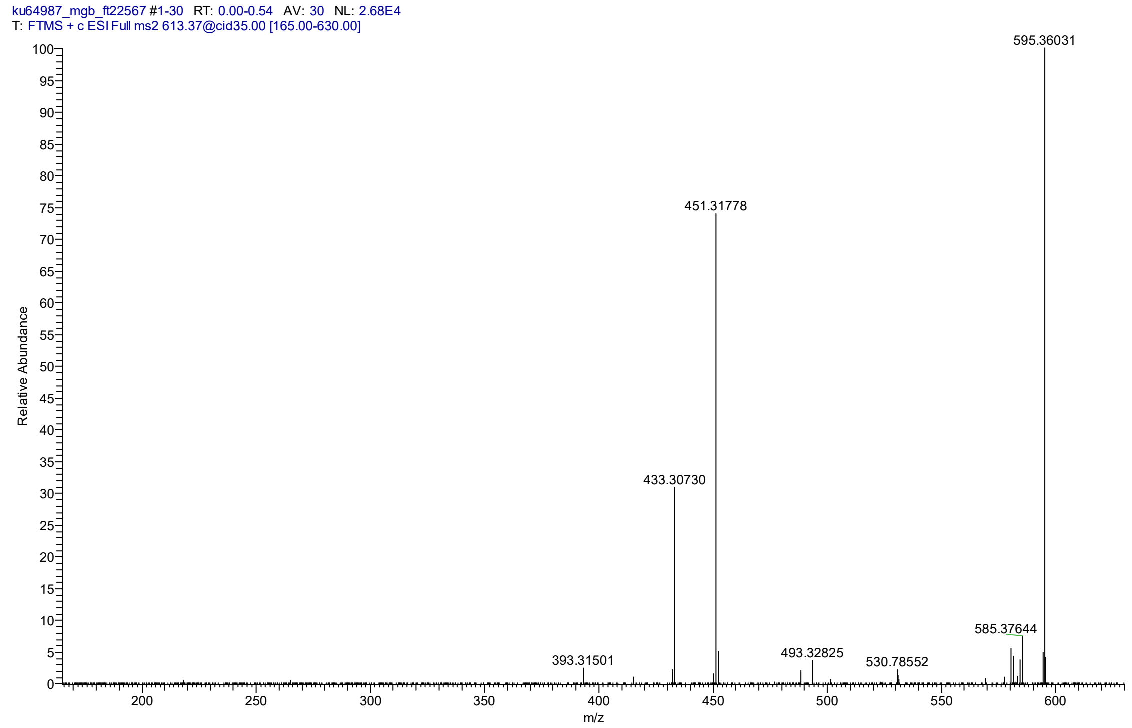

Figure S30. MSMS spectrum of compound **8**

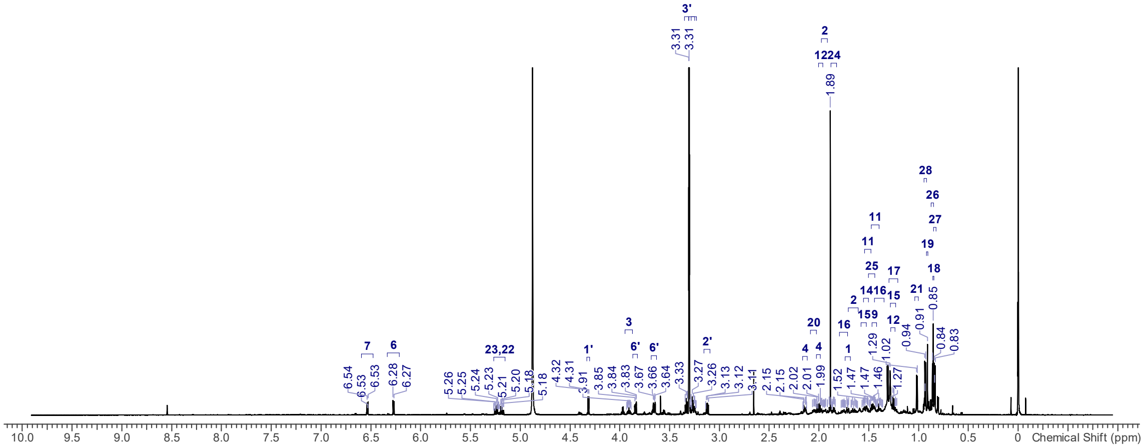

Figure S31. 800 MHz ^{1}H spectrum of compound **8** in MeOD.

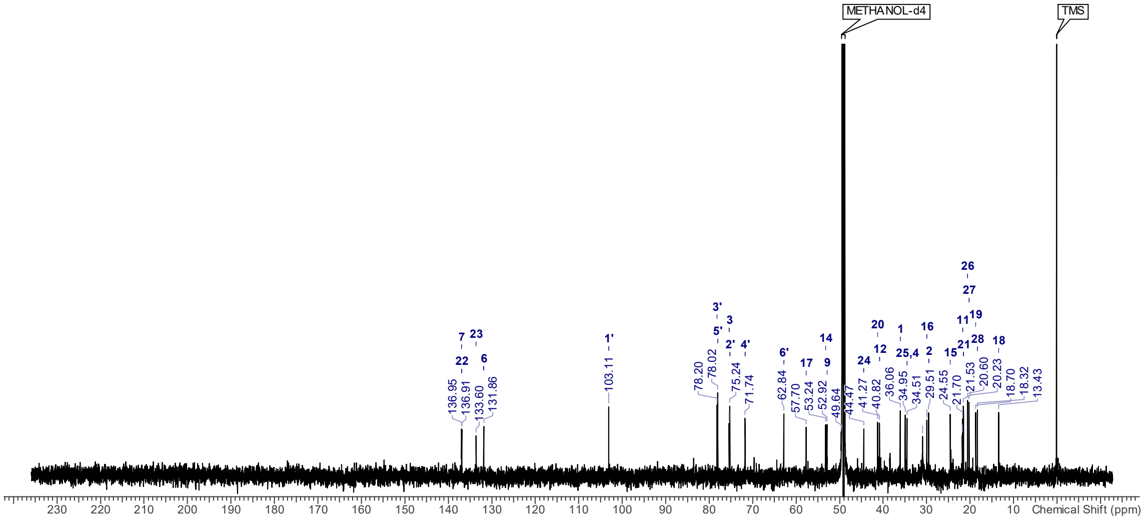

Figure S32. 201 MHz ^{13}C NMR spectrum of compound **8** in MeOD

Spectra and spectral data on compound 9

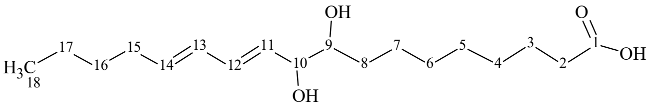

^{1}H NMR (800 MHz, METHANOL-d_4) δ = 6.21 (1H, dd, J = 15.4 Hz, J = 10.4 Hz, H-12), 6.03 - 6.10 (1H, m, H-13), 5.69 (1H, dt, J = 15.1 Hz, J = 6.9 Hz, H-14), 5.64 (1H, dd, J = 15.3 Hz, J = 7.2 Hz, H-11), 3.91 (1H, ddd, J = 7.1 Hz, J = 5.0 Hz, J = 0.8 Hz, H-10), 3.48 (1H, ddd, J = 8.1 Hz, J = 4.9 Hz, J = 2.9 Hz, H-9), 2.27 (2H, t, J = 7.5 Hz, H-2), 2.09 (2H, qd, J = 7.2 Hz, J = 1.2 Hz, H-15), 1.56 - 1.64 (2H, m, H-3), 1.46 - 1.56 (1H, m, H-7), 1.25 - 1.43 (13H, m, H-4, 5, 6, 7, 8, 16, 17), 0.91 (3H, t, J = 7.3 Hz, H-18)

^{13}C NMR (201 MHz, METHANOL-d_4) δ = 177.9 (C-1), 135.9 (C-14), 133.8 (C-12), 131.4 (C-13), 131.3 (C-11), 77.1 (C-10), 75.9 (C-9), 35.2 (C-2), 33.9 (C-8), 33.5 (C-15), 32.8 (C-16), 30.8 (C-6), 30.5 (C-5), 30.3 (C-4), 27.0 (C-7), 26.3 (C-3), 23.4 (C-17), 14.4 (C-18)

HRMS: M+H+NH_3=330.26398 (delta=0.3 ppm; $C_{18}H_{36}O_4N$). HR-ESI-MS-MS (CID=45%; rel. int. %): 312(14); 295(100); 277(3).

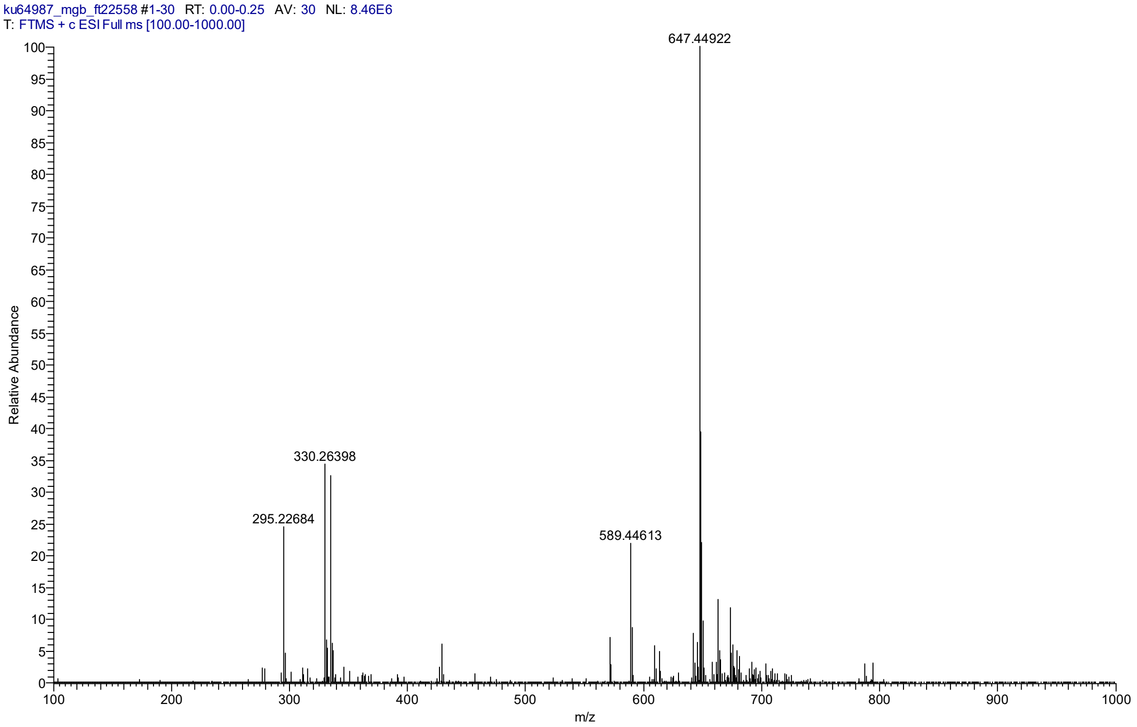

Figure S33. HRESI-MS spectrum of compound **9**

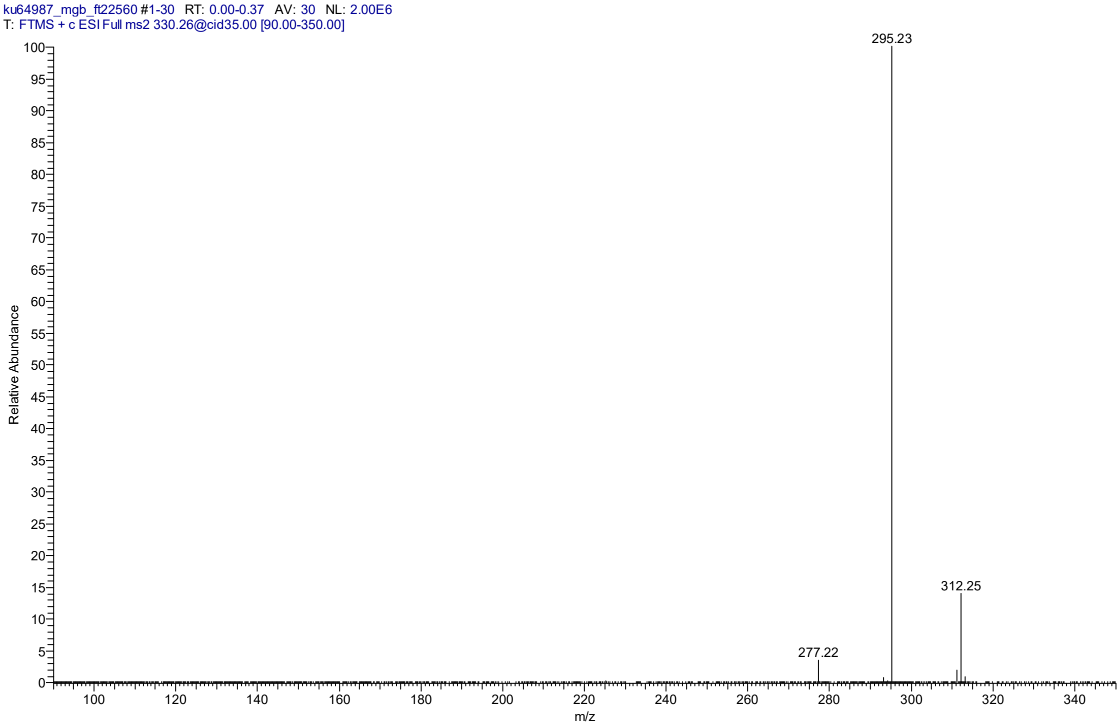

Figure S34. MSMS spectrum of compound **9**

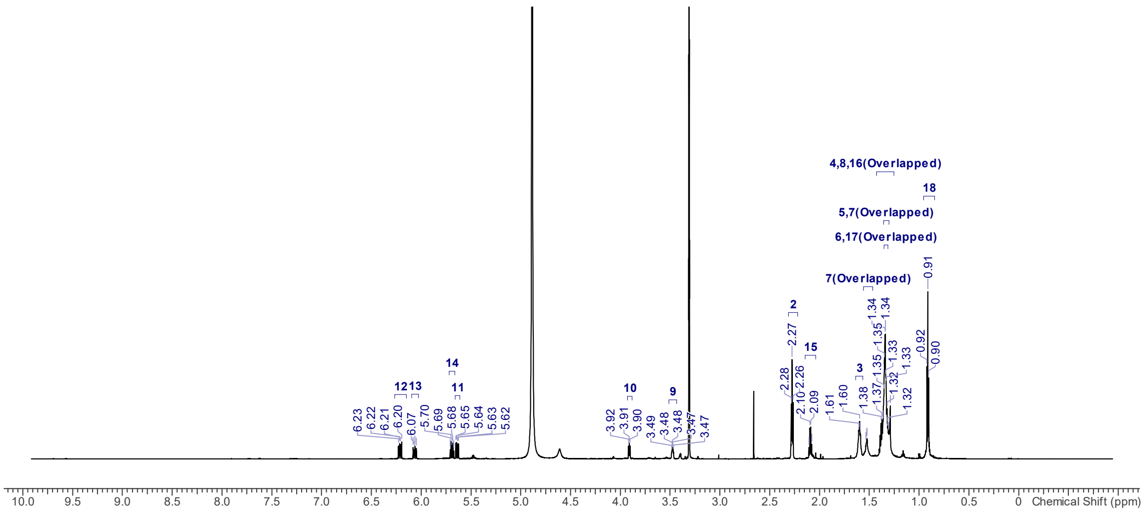

Figure S35. 800 MHz 1H spectrum of compound **9** in MeOD

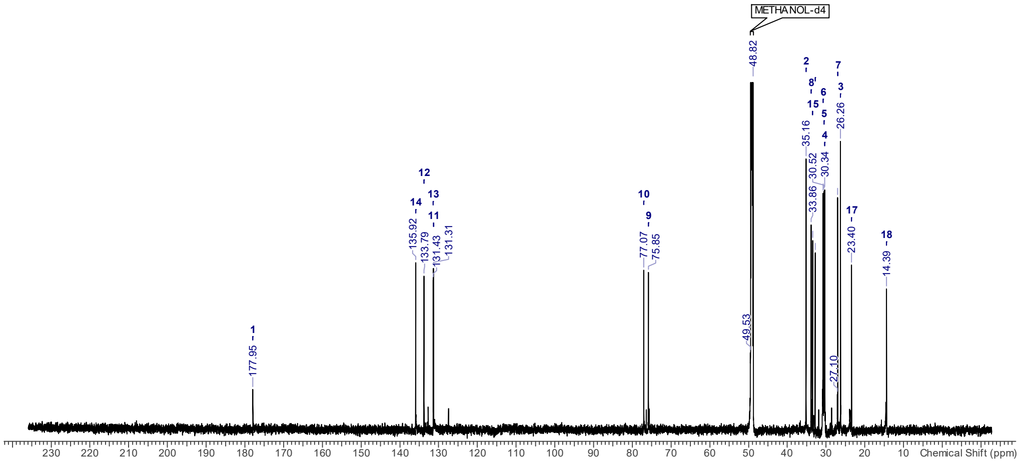

Figure S36. 201.1 MHz ^{13}C NMR spectrum of compound **9** in MeOD.
